# Supplementary material for: Catch bond models may explain how force amplifies TCR signaling and antigen discrimination
Source: Nat Commun. 2023 May 5;14:2616. doi: 10.1038/s41467-023-38267-1 (PMC10163261; doi:10.1038/s41467-023-38267-1)
Supplement: Supplementary file 1 — Supplementary Information [file 41467_2023_38267_MOESM1_ESM.pdf]

Supplementary Information

Catch bond models may explain how force amplifies TCR signaling and antigen discrimination

Hyun-Kyu Choi[1,2], Peiwen Cong[1,2], Chenghao Ge [1,2,11], Aswin Natarajan[3,4], Baoyu Liu[1,2,12], Yong Zhang[5,6,7], Kaitao Li[1,2], Muaz Nik Rushdi[1,2,13], Wei Chen[8,9], Jizhong Lou[5,6,7], Michelle Krogsgaard[3,4], and Cheng Zhu[1,2,10*]

*Corresponding author. Email: cheng.zhu@bme.gatech.edu

This PDF file includes:

Supplementary Model Derivations
Supplementary Figures. 1 to 12
Supplementary Tables 1 to 6
References (1 to 24)

Supplementary Methods

Supplementary Model Derivations

A. Kinetic model for TCR–pMHC-I bonds
  A.1. Derivation of the force-induced energy change $\delta_1\gamma(F)$
  A.2. Simplifying assumptions and reducing parameters
  A.3. Defining the dissociation coordinate
  A.4. Model applications, curve-fitting strategies, and biological relevance
  A.5. Class I model constraints
B. Kinetic model for TCR–pMHC-II bonds
  B.1. Development, validation, and characterization
  B.2. Class II model constraints
C. A general biophysical limit of model parameters

Supplementary Figures

Supplementary Fig. 1 | Fitting class I model to data of TCR–pMHC-I bond lifetime *vs* force
Supplementary Fig. 2 | Correlation of class I model parameters with biological activity and TCR bond type
Supplementary Fig. 3 | Hydrogen (H) bond distributions at the TCR–pMHC-I bonding interface
Supplementary Fig. 4 | Pulling and tilting constraints of class I model
Supplementary Fig. 5 | Determining the number of clusters in parameter space
Supplementary Fig. 6 | Lack of correlations between best-fit parameters of the two-pathway model and T cell function
Supplementary Fig. 7 | Characterization of the energy landscape of TCR–pMHC-II dissociation
Supplementary Fig. 8 | Correlation of metrics of TCR–pMHC-II bond lifetime *vs* force curves with T cell biological activity
Supplementary Fig. 9 | Comparing fitting curves of class I and class II models for the same data
Supplementary Fig. 10 | Relative goodness-of-fit of the class I and II models and the two-pathway model to class I and class II data
Supplementary Fig. 11 | Examples of gating strategy for flow cytometry
Supplementary Fig. 12 | Comparison of pMHC interactions with cell surface and purified TCRs

Supplementary Tables

Supplementary Table 1 | Summary of model constants
Supplementary Table 2 | Finding the best-fit parameters for OT1 TCR–OVA:H2-K^ba3A2 bond
Supplementary Table 3 | TCR–pMHC-I bond summary and their best-fitting parameters.
Supplementary Table 4 | Two-pathway model summary and their best-fitting parameters.
Supplementary Table 5 | Finding the best-fit parameters for the 3.L2 TCR–Hb:I-E^k bond.
Supplementary Table 6 | TCR–pMHC-II bond summary and their best-fit parameters.

Supplementary References

Supplementary Model Derivations

A. Kinetic model for TCR–pMHC-I bonds

A.1. Derivation of the force-induced energy change $\boldsymbol{\delta_\mathrm{l}\gamma(F)}$

The purpose of this subsection is to provide detailed derivation of the force-induced change in the energy landscape, or the work done by force as the TCR–pMHC complex is stretched at the two ends from the bound state to the transition state until dissociation (Fig. 2a, b). Mathematically, $\delta_\mathrm{l}\gamma(F)$ is an integral over a force range $0 \le f \le F$, where F is the level of force under which the kinetic rate is being evaluated (Eq. 2). The integrand $\delta_\mathrm{z}(f)$ is the projection on the force direction of the length change of the TCR–pMHC structure relative to its bound state during dissociation. Subtraction of this work from the interaction free energy tilts the energy landscape that governs the off-rate (Eq. 1, Fig. 1b). To calculate molecular stretch, we assume the TCR–pMHC complex to behave as a system of semi-rigid bodies of globular domains connected by semi-flexible polymers (Fig. 2b). As such, the total length change includes three components: 1) extension of individual globular domains, 2) various domain rotations about hinges, and 3) unfolding of secondary structures at specific regions.

For a globular domain without unfolding, the force-extension relationship is described by the three-dimensional freely-jointed chain model[1]:

$$d_\mathrm{i}(f) = d_\mathrm{i,c}\left\{\coth\left[f d_\mathrm{i,c}/k_\mathrm{B}T\right] - \frac{k_\mathrm{B}T}{f d_\mathrm{i,c}}\right\}\left(1 + \frac{f}{E_\mathrm{d}}\right), \qquad \mathbf{(5)}$$

where d_i is end-to-end distance of the ith-domain, k_B is the Boltzmann constant, T is absolute temperature, f is force, and E_d ~ 100 pN is the elastic modulus of the folded globular domain[2]. The present work considers domain extension of the whole TCR–pMHC-I complex (d_N) and three of its parts: the MHC α_3 domain ($d_{\alpha3}$), bonding interface that includes the MHC $\alpha_1\alpha_2$ domains bound to the TCR V$\alpha\beta$ domains ($d_\mathrm{B.I}$), and the TCR C$\alpha\beta$ domains ($d_{\mathrm{C}\alpha\beta}$). Their contour lengths, $d_\mathrm{i,c}$, have well-defined values depending on the TCR–pMHC complex in

question, which are summarized in Supplementary Table 1. For example, $d_{\mathrm{N,c}}$ = 12.7 and 10.9 nm for 2C TCR complexed with H2-K^b (PDB codes 2CKB, 1MWA, and 1G6R) and H2-L^{d}m31 (2E7L), respectively, and 12.3 nm for 1G4 TCR complexed with HLA-A2 (2BNR and 2BNQ). 12.7 nm and 12.5 nm were used for P14 TCR (5M00) and NP1-B17 TCR complexed with H2-D^b (5SWZ), respectively. We also choose 11.6 nm as a reasonable guess value for the N15 TCR/OT1 TCR–p:H2-K^b complex (because average $d_{\mathrm{N,c}}$ across 33 structures of TCR-pMHC class I complexes is 11.6 ± 1 nm (mean ± sd)[3]).

To calculate the work $\delta_1\gamma(F)$, we project the above domain extensions onto the direction of force, which is taken as the *z* direction (Fig. 2b) using two angles between the *z* axis and: 1) the normal direction of the bonding interface (θ) and 2) the line connecting the C- and N-termini of the MHC-I α_3 domain excluding any unfolded residues (φ):

$$z_{\mathrm{N}}(f) = d_{\mathrm{N}}(f)\,, \tag{6a}$$

$$z_{\alpha3}(f) = \sqrt{d_{\alpha3}^2(f) - \left\{\left[d_{\mathrm{N}}(f) - d_{\mathrm{C\alpha\beta}}(f)\right]\sin\theta - d_{\alpha3}(f)\sin(\varphi+\theta)\right\}^2}\,, \tag{6b}$$

$$z_{\mathrm{B.I}}(f) = d_{\mathrm{B.I}}(f) \equiv \left[d_{\mathrm{N}}(f) - d_{\mathrm{C\alpha\beta}}(f)\right]\cos\theta - d_{\alpha3}(f)\cos(\varphi+\theta)\,, \tag{6c}$$

$$z_{\mathrm{C\alpha\beta}}(f) = d_{\mathrm{C\alpha\beta}}(f)\,, \tag{6d}$$

Note that θ is a model parameter as it describes the tilting of the bonding interface as part of the force-induced conformational change, whereas φ is a model constant is measured from the crystal structure (Supplementary Table 1, see A.2 below).

We assume that partial unfolding in the molecular complex may occur at connecting regions of globular domains, in particular, the $\alpha_1\alpha_2$–α_3 joint of the MHC-I and the Vα-Cα interdomain joint of the TCR (Fig. 2a). The former may be caused by dissociation of the noncovalent $\alpha_1\alpha_2$–β_{2m} interdomain bond, which shifts the mechanical load originally borne by this bond to the $\alpha_1\alpha_2$–α_3 hinge, resulting in its partial unfolding, as observed in SMD

simulations[4] (Fig. 2a). Similarly, $\alpha_1\alpha_2$–β_{2m} dissociation results in tilting of the bonding interface and load shifting from the Vβ-Cβ joint to the Vα–Cα joint, leading to partial unfolding of the latter joint (Fig. 2a). The unfolded polypeptides are flexible and can bear only tension but not moment, ensuring that their extension is along the direction of force, i.e., the *z* axis.

The force-extension relationship of the unfolded polypeptides can be described by an extensible worm-like chain (eWLC) model[5]:

$$\frac{f l_{\mathrm{p}}}{k_{\mathrm{B}}T} = \frac{1}{4\left(1 - z_{\mathrm{p}}/(n l_{\mathrm{c}})\right)} - \frac{1}{4} + \frac{z_{\mathrm{p}}}{n l_{\mathrm{c}}} - \frac{f}{K_{\mathrm{p}}} + \sum_{\mathrm{j}=1}^{\mathrm{j}\le 7} a_{\mathrm{j}} \left(\frac{z_{\mathrm{p}}}{n l_{\mathrm{c}}} - \frac{f}{E_{\mathrm{p}}}\right)^{\mathrm{j}}, \quad \textbf{(7)}$$

where z_{p} is the extension of the unfolded coil under force with the subscript p indicating unstructured polypeptide, $l_{\mathrm{c}} = 0.36$ nm and $l_{\mathrm{p}} = 0.39$ nm are the average contour length and persistence length per unfolded amino acid, respectively[6-8], $E_{\mathrm{p}} \sim 50$ μN is the elastic modulus of polypeptides[8], a_{j} are polynomial coefficients for the improved approximation, and n is the number of constituent amino acids in the unfolded polypeptide. In particular, we denote the respective numbers of amino acids in the unfolded MHC-I $\alpha_1\alpha_2$–α_3 and TCR Vα-Cα joints to be $n_{\mathrm{p,MHC}}$ and $n_{\mathrm{p,TCR}}$. Eq. 7 defines z_{p} as a function of *f*, which can be solved numerically to express in an explicit form: $z_{\mathrm{p}}/n l_{\mathrm{c}} = z_{\mathrm{u,p}}(f)$ = the extension per unit contour length for the polypeptide under force *f*.

Thus, the length of the TCR–pMHC complex at the transition state is (Fig. 2b):

$$z(f) = z_{\alpha 3}(f) + n_{\mathrm{p,MHC}} l_{\mathrm{c}} z_{\mathrm{u,p}}(f) + z_{\mathrm{B.I}}(f) + n_{\mathrm{p,TCR}} l_{\mathrm{c}} z_{\mathrm{u,p}}(f) + z_{\mathrm{C\alpha\beta}}(f), \quad \textbf{(8)}$$

Since we do not have prior knowledge about either number of unfolded amino acids, we will evaluate their sum $n^* = n_{\mathrm{p,TCR}} + n_{\mathrm{p,MHC}}$ from curve-fitting of our model to the experimental data (see below). Since $d_{\mathrm{N}}(f)$ is the length of the TCR–pMHC complex at the bound state (Fig.

2b), we have $z(f;0) = d_N(f)$. Finally, the integrand on the right-hand side of Eq. 3 can be written as

$$\delta_z(f) = z_{\alpha 3}(f) + z_{B.I}(f) + z_{C\alpha\beta}(f) + n^* l_c z_{u,p}(f) - d_N(f)\,, \quad \textbf{(9)}$$

A.2. Simplifying assumptions and reducing parameters

The purpose of this subsection is to provide the details omitted in the main text of how the model parameters are reduced to the smallest possible set and the underlying simplifying assumptions. To begin, we take the φ values from PDB for the specific TCR–pMHC interactions in question (Supplementary Table 1). We assume that the φ value remains constant during forced dissociation due to their small range, *e.g.*, φ = 12.7° and 5° for 2C TCR complexed with H2-K^b (PDB codes 2CKB, 1MWA, and 1G6R) and H2-L^{d}m31 (2E7L), respectively, and 13.3° for 1G4 TCR complexed with HLA-A2 (2BNR and 2BNQ), 15° for both P14 TCR (5M00) and NP1-B17 TCR complexed with H2-D^b (5SWZ). We also chose 23.5° as a reasonable guess value for the N15 TCR/OT1 TCR–p:H2-K^b complex. Additionally, due to the semi-rigid approximation, the thickness of the constant domain of TCR α- and β-subunit ($d_{C\alpha\beta}$) is also treated as constant for each construct (Supplementary Table 1). This leaves only two structural parameters ($d_{\alpha 3}$, θ) in our model to be evaluated by curve-fitting to data. Noting that the structure-based force function $\gamma(F;\delta_l)$ scales with the characteristic extension change per unit change of molecular length such that $\delta_l \gamma(F) = \int_0^F \delta_z(f)df$, the dissociation rate of TCR–pMHC-I bond can be written as:

$$k(F) = k_0 \sqrt{1 - \frac{2\int_0^F \delta_z(f)df}{3\Delta G_0^*}}\, e^{\frac{\Delta G_0^*}{k_B T}\left(1-\left(1-\frac{2\int_0^F \delta_z(f)df}{3\Delta G_0^*}\right)^{3/2}\right)}\,, \quad \textbf{(10)}$$

where $\delta_z(f)$ is given by Eq. 9.

To further reduce model parameters, we make additional assumptions as discussed in the main text. It is well-known that the fractions of free-energy change in biological interactions

in liquid, such as unfolding and refolding of proteins, unbinding and rebinding of receptor–ligand bonds, and unzipping and rezipping of RNA or DNA, are small because of their limited dynamic transition time[9-12]. Such a rate limit results from the nature of biological interactions, *e.g.*, polar/non-polar interactions, hydrophobic interactions, and charged interactions, which typically yield finite range of transition kinetics. This enables to roughly estimate the free-energy barrier as $\Delta G_0^* \sim \ln(k_\mathrm{w}/k_0)$ where $k_\mathrm{w} \sim 10^6$ s^{-1} known as the prefactor[9-12].

A.3. Defining the dissociation coordinate

The purpose of this subsection is to derive an operational way to determine the dissociation coordinate variable δ_l used in the main text. We note that the total number of unfolded amino acids n^* is zero at the bound state before unfolding occurs, increases monotonically during progressive unfolding along the dissociation path, and reaches maximum at the dissociation point. Because n^* is not known *a priori*, it must be treated as a fitting parameter similar to $d_{\alpha 3}$, θ, and δ_0^*. Since δ_l is the contour length change along the dissociation path, we wish that δ_l approaches its upper bound δ_0^* and depends on force implicitly through the model parameters n^*, $d_{\alpha 3}$, and θ, as given below:

$$\delta_\mathrm{l} = n^* l_\mathrm{c} + \Delta(d_{\alpha 3}, \theta) \rightarrow \delta_0^* \,, \qquad \textbf{(11)}$$

where $\Delta(d_{\alpha 3}, \theta)$ is the difference of the contour lengths except for the partially unfolded regions. Thus, Eq. 11 provides a constraint for δ_l instead of introducing another model parameter. $d_{\alpha 3}$ and θ are determined for each n^* during the model fitting that searches for parameters to enable $\delta_\mathrm{l} \rightarrow \delta_0^*$. Small errors, which may occur for the various contributions to Δ (red lines in Fig. 2a labeled as force transmission lines), can be identified using a pair of $(d_{\alpha 3}, \theta)$ values and the crystal structure for each complex. Specifically, average differences $\langle \Delta \rangle$ are - 0.8 nm for the strong catch bond (SC) group ($d_{\alpha 3} > 3$ nm, $\theta > 30°$), -0.4 nm for the weak catch bond (WC) group (1 nm $< d_{\alpha 3} <$ 3 nm, 8°$< \theta <$ 30°), and 0 nm for slip-only bond

(SO) group ($d_{\alpha 3} < 1$ nm, $\theta < 8°$), respectively (see Fig. 4e-i and associated text for the definitions of SC, WC, and SO groups). It has been well established that the contour length of a single amino acid is ~0.4 ± 0.02 nm/a.a[7,13,14], implying that the model has a resolution of 2 amino acids. We further note that, even without conformational change, it is possible for the slip-only bond group to have 3 unfolded amino acids due to the limited resolution of the model. Finally, the best-fit model parameters can be determined by finding the subset of best-fit parameters among possible n^* values that match the contour length change δ_l to the free-energy well width at zero-force δ_0^*, *i.e.*, finding n^* such that $\delta_l(n^*, d_{\alpha 3}, \theta) \to \delta_0^*$ (see Supplementary Table 2). Under this condition, $\int_0^F \delta_z(f)df = \delta_l \gamma(F) \to \delta_0^* \gamma(F)$ and Eq. 10 becomes identical to Eq. 2.

A.4. Model applications, curve-fitting strategies, and biological relevance

The purpose of this subsection is to outline the procedures of applying our model to experiments. Our procedures include four steps: 1) examine how the model parameters control the model behaviors, 2) fit the model-predicted reciprocal off-rate (Eq. 10) to the experimental bond lifetime *vs* force data, 3) construct the energy landscape and investigate its properties based on the parameters evaluated in part 2, 4) elucidate the biological relevance of the model parameters.

To examine how the model parameters control the model behaviors, we varied one parameter while keeping others constant. For example, to investigate the effect of varying the titling angle θ, we kept the other parameters constants (*i.e.*, $n^* = 7$, and $\delta_0^* = 2$ nm, $k_0 = 3$ s^{-1} and $\Delta G_0^* = 12.7\ k_BT$), Also kept constant were several structural constants ($\varphi = 15°$, $d_N = 12$ nm and $d_{C\alpha\beta} = 3.5$ nm) introduced in section A.1. Of note, $d_{\alpha 3}$ varies as θ changes because of a pulling constraint (see section B.1). With a fixed θ (*i.e.*, 30°) the effect of molecular extension at zero force was investigated by varying extension from 0.5-3.5 nm. These were

selected by the average values of actual fitting results, which had been tested and confirmed as reasonable.

The free-energy landscape can be constructed by substituting the best-fit model parameters into the following equations[14]:

$$\Delta G^*(F) = \Delta G_0^* \left(1 - \frac{2\delta_0^*\gamma(F)}{3\Delta G_0^*}\right)^{3/2}, \quad \textbf{(12a)}$$

$$\delta^*(F) = \delta_0^* \left(1 - \frac{2\delta_0^*\gamma(F)}{3\Delta G_0^*}\right)^{1/2}, \quad \textbf{(12b)}$$

Thus, by using model parameters, the dissociation state coordinates relative to the bound state coordinates in the free-energy *vs* dissociation coordinate space can be defined as functions of force. Note that this force-induced change of the barrier height should be under the condition of small perturbation such that $|-\delta_0^*\gamma(F)| < \Delta G_0^*$. Since our fitting results show that the average free-energy barrier height at zero force is ~12 k_BT ($\langle \Delta G_0^* \rangle$), the force range corresponding to a change of the barrier height of <10 k_BT is reasonable for each dataset, *i.e.,* force range corresponding to energy barrier heights in the range of 2 $k_BT < \Delta G_0^* <$ 22 k_BT.

Firstly, to fit the model-predicted reciprocal off-rate (Eq. 10) to the experimental bond lifetime *vs* force data, all four parameters were changed simultaneously to search for the minimum of the chi squared error. Second, every fitting curve was then determined by the parameter set that has the closest δ_1 value to δ_0^* (described as Eq. 11 in section A.3 and Supplementary Table 2). The fitting uncertainty of the best-fit parameters (or their ranges) were calculated by the differences between the previously obtained parameter set and parameter set from fitting to Mean ± SEM of bond lifetimes with the lowest RSS and the lowest Chi-square. Of the 55 datasets analyzed, only one has 4 data points and this dataset shows slip bond; as such, is governed by two fitting parameters because the other two parameters are nearly zero. All other datasets have 6-10 data points; therefore, over-fitting is not a problem.

To elucidate the biological relevance of the model parameters θ, n^*, $d_{\mathrm{C\alpha\beta}}$, δ_0^*, and ΔG_0^*, we examine their changes with varying bond lifetime *vs* force data obtained from different TCR and pMHC interactions that induce a wide range of biological responses. Finding correspondence between a group of model parameters individually and/or collectively with the biological response would be considered as support for the biological relevance of the model, because such correspondence suggests that the model can discriminate different TCR–pMHC interactions that result in differential T cell functions.

A.5. Class I model constraints

The purpose of this subsection is to check whether the model parameters obtained from data fitting are consistent with the constraints to which the model is subjected. Our experiments applied tensile force through the two ends of the TCR–pMHC complex, such that the force direction would always align with the line connecting the C-termini of the respective TCR and MHC molecules during dissociation, giving rise to the so-called pulling constraint. To formulate this pulling constraint in our model, we note that the pulling line is maintained so that the coordinate perpendicular to the force direction is invariant. As depicted in Supplementary Fig. 4a, several angle and length variables can be related using model parameters and structural constants by:

$$\left(d_N - d_{\mathrm{C\alpha\beta}} - d_{\alpha 3}\frac{\cos(\varphi+\theta)}{\cos\theta}\right)\sin\theta = d_{\alpha 3}\left(\frac{\sin\varphi}{\cos\theta} + \cos\left(2\psi + \varphi + \theta - \frac{\pi}{2}\right)\right), \qquad \textbf{(13a)}$$

where ψ is the angle in an isosceles triangle constructed by rotating the α3 domain. By assuming that the α3 domain would be aligned with force, we estimate that the angle in the last term is near 90°, *i.e.*, $2\psi + \varphi + \theta - \frac{\pi}{2} \approx \frac{\pi}{2} - \delta$ where $\delta \ll \frac{\pi}{2}$. Under this small angle assumption, Eq. 13a can be approximated by:

$$d_{\alpha3} \approx \frac{d_N - d_{C\alpha\beta}}{\cot\theta \sin\varphi + \cos\varphi + \sin\delta / \sin\theta}\ , \qquad \textbf{(13b)}$$

Upon inversing Eq. 13b, we found that the tilting angle θ is a function of the end-to-end distance of the α3 domain, *i.e.,* $\theta = f(d_{\alpha3})$. Setting $\delta = 25°$, which seems reasonable as it approximates the maximum value of φ, the structural parameters obtained by fitting are scattered in-between two black curves on the $d_{\alpha3} - \theta$ plane marked as pulling constraint: [$d_N = 13.5$ nm, $d_{C\alpha\beta} = 2.75$ nm, $\varphi = 0°$] and [$d_N = 10.5$ nm, $d_{C\alpha\beta} = 4.25$ nm, $\varphi = 25°$] (Fig. 4f).

Another constraint is the tilting constraint resulted from the asymmetric unfolding and stretching of the interdomain links between the TCR constant and variable domains. This constraint is introduced in the model to account for the potential regulatory effect of the FG-loop on catch bond. Notwithstanding the total number of unfolded amino acids n^* can be determined by the validation procedure demonstrated in A.2, its breakdown into the number of unfolded amino acids for MHC ($n_{p,MHC}$) and TCR ($n_{p,TCR}$) remains undetermined. To do this, known structures from *PDB* were used to determine $n_{p,MHC}$ first. Briefly, by matching $d_{\alpha3}$ (Supplementary Fig. 4b, c) with the end-to-end distance between C-terminal end of α3 domain and certain point following known *PDB* structure, the exact starting position of partial unfolding in MHC can be found following additional assumption that unfolding starts from C-terminal end of α2 domain towards α3 domain (Supplementary Fig. 4b). Thus, $n_{p,TCR}$ can be simply calculated as $n_{p,TCR} = n^* - n_{p,MHC}$. Upon combining all information, the tilting angle (θ_{TCR}) of variable domains of TCR can be described by simple trigonometrical function:

$$\theta_{TCR} \approx \tan^{-1}\left(\frac{n_{p,TCR} l_c}{W_{TCR}}\right), \qquad \textbf{(14)}$$

where W_{TCR} is width of two interdomain hinges of the TCR α- and β-subunits measured from the crystal structures (Supplementary Fig. 4a, tilting constraint). In this work we use $W_{TCR} = 3.7 \pm 0.3$ nm as a representative width due to structure-to-structure variations. Thus, by

comparing the tilting angle of the bonding interface θ (model parameter) to the tilting angle of the TCR θ_{TCR} (derived from another model parameter $n_{\mathrm{p,TCR}}$ and structural constants W_{TCR} and l_{c}), we can check the validity of tilting constraint using linear regression in the θ *vs* θ_{TCR} plot (Supplementary Fig. 4d).

B. Kinetic model for TCR–pMHC-II bonds

B.1. Development, validation, and characterization

The purpose of this subsection is to present details of the development, validation, and characterization of the TCR–pMHC-II catch bond model omitted in the main text for simplicity, in a similar fashion as the TCR–pMHC-I catch bond model described in Section A. The two models share exact the same framework but have different detailed form of the characteristic extension change $\delta_{\mathrm{z}}(f)$ (Eq. 9). Comparing to the TCR–pMHC-I complex, the TCR–pMHC-II complex has different docking domains and pulling geometries (one *vs* two transmembrane domains on both TCR and MHC). For this reason, we assume that the force-induced bonding interface tilting angle (θ) would be much smaller in the TCR–pMHC-II than TCR–pMHC-I complex. The extension at the bound state can be defined as the end-to-end distance between both end-points identified by crystal structures (E8: 2IAM, 2IAN and 2B4 (as the substitution of 3.L2): 6BGA, 3QIB):

$$z(f;0) = d_{\mathrm{N}}(f) + d_{\mathrm{linker}}(f)\,, \qquad \textbf{(15)}$$

where d_{N} is set to be 12.3 nm based on the crystal structures and $d_{\mathrm{linker}} \approx 9.4$ nm represents the linker (*e.g.*, a leucine zipper) engineered at the C-termini of soluble pMHC-II constructs to stabilize both the MHC α- and β-subunits, which is often used in experiments for measuring TCR–pMHC-II catch bonds. To account for domain rotation resulted from partial unfolding inside the TCR–pMHC-II complex, we introduce one more variable, φ, as the tilting angle of

the TCR constant domains. Using the tilting constraint similarly to that used in the class I model, this angle can be approximately described by structural parameters ($d_{\mathrm{B.I}}$, θ) and model constants (see subsection B.2). In short, each component in the right-hand side of Eq. 15 can be expressed by the model parameters ($d_{\mathrm{B.I}}$, θ, n^*), and model constants as follows.

$$z_{\mathrm{B.I}}(f) = \left(d_{\mathrm{B.I}}(f) + z_{\mathrm{p,MHC}}(f)\right)\cos\theta\ , \qquad \textbf{(16a)}$$

$$z_{\mathrm{C\alpha\beta}}(f) = \left(d_{\mathrm{C\alpha\beta}}(f) + z_{\mathrm{p,TCR}}(f)\right)\cos\varphi\ , \qquad \textbf{(16b)}$$

$$z_{\mathrm{linker}}(f) = d_{\mathrm{linker}}(f)\cos\theta\ , \qquad \textbf{(16c)}$$

where $z_{\mathrm{p,MHC}}(f)$ and $z_{\mathrm{p,TCR}}(f)$ are respective extensions of unfolded polypeptides given by $n_{\mathrm{p,MHC}} l_{\mathrm{c}} z_{u,p}(f)$ and $n_{\mathrm{p,TCR}} l_{\mathrm{c}} z_{u,p}(f)$, respectively, $d_{\mathrm{B.I}}$ is the length of structure consisting of the MHC and the TCR variable domains, and other parameters defined previously. Finally, the rate coefficient of the TCR–pMHC-II dissociation can be developed by employing the same framework (see Eq. 10). However, when applying the model to experimental data, we can use the constraints of the TCR–pMHC-II interaction to make the model much simpler (see section B.2).

Validation of the class II model follows exactly the same procedure as that used in the validation of the pMHC I model, which is done by checking self-consistent through the definition of the reaction coordinate. By varying the $n_{\mathrm{p,MHC}}$ from 0 to 10 (see details explained in section B.2), the molecular extension at zero force (δ_0^*) can be estimated by using the contour length-change (δ_{l}) along the dissociation coordinate:

$$\delta_{\mathrm{l}} = n_{\mathrm{p,MHC}} l_{\mathrm{c}} + \Delta \rightarrow \delta_0^*\ , \qquad \textbf{(17)}$$

where $\Delta(d_{\mathrm{B.I}}, \theta) < 0.2$ nm because $\theta < 10°$. The best-fit model parameters can be determined by finding a subset of best-fit parameters among possible $n_{\mathrm{p,MHC}}$ values that match the contour

length-change along the dissociation coordinate (δ_l) to the width of the free-energy well at zero force (δ_0^*), *i.e.*, finding $n_{\mathrm{p,MHC}}$ such that $\delta_\mathrm{l}(n_{\mathrm{p,MHC}}) \to \delta_0^*$ (see Supplementary Table 5).

To characterize the class II model, we examine the model predictions by varying the model parameters one by one while fixing the others as constants. For example, to investigate force-induced bonding interface titling, we fixed the other model parameters (*i.e.*, $n^* = 4$, $\delta_0^* =$ 1.2 nm, $k_0 = 10$ s^{-1}, and $\Delta G_0^* = 11.5\ k_\mathrm{B}T$) and structural constants ($d_\mathrm{N} = 12.3$ nm, $d_{\mathrm{B.I}} = 8.5$ nm, and $d_{\mathrm{linker}} = 9.4$ nm for pMHC-II constructs that have linkers). As another example, we fixed $\theta = 0°$ or 3° and examined the effect of the molecular extension at zero force by varying δ_0^* from 0.3-1.8 nm. The constants used for model characterization were selected by their averages from the corresponding values used to fit actual experiments. The parameters for free-energy landscape construction, the energy barrier height (ΔG^*) and energy well width (δ^*) as functions of force, are given by Eq. 12, exactly the same as the pMHC I model.

Fitting of class II model to experimental data and examination of the biological relevance of the best-fit parameters were done the same way as the class I model.

B.2. Class II model constraints

The purpose of this subsection is to describe the constraints of the class II model, which share similar ideas to those of the class I model (*e.g.*, pulling and tilting constraints) but differ in their specific expressions. To formulate the pulling constraint, we again used the fact that the pulling force direction must aligns with the line connecting to the C-termini of the TCR and pMHC molecules so that the coordinate perpendicular to the force direction is invariant. Using model parameters and structural constants, this pulling constraint can be written as:

$$\left(d_{\mathrm{B.I}} + z_{\mathrm{p,MHC}} + d_{\mathrm{linker}}\right) \sin\theta = \left(d_{\mathrm{C\alpha\beta}} + z_{\mathrm{p,TCR}}\right) \sin\varphi\ , \quad \textbf{(18a)}$$

which can be solved for $z_{\mathrm{p,TCR}}$ explicitly:

$$z_{\mathrm{p,TCR}} = \left(d_{\mathrm{B.I}} + z_{\mathrm{p,MHC}} + d_{\mathrm{linker}}\right)\frac{\sin\theta}{\sin\varphi} - d_{\mathrm{C\alpha\beta}} \ , \tag{18b}$$

By combining Eq. 16 and 18, the total extension (z) can be calculated as the sum of all component extensions ($\sum_i z_i$) at dissociation ($\delta_l > 0$):

$$z(f;\delta_l) = \left(d_{\mathrm{B.I}}(f) + z_{\mathrm{p,MHC}}(f) + d_{\mathrm{linker}}(f)\right)(\cos\theta + \sin\theta\cot\varphi) \ , \tag{19}$$

This equation states that only the number of unfolded amino acids in MHC ($n_{\mathrm{p,MHC}}$) affects extension change during transition. The total number of unfolded amino acids from both TCR and MHC can be estimated by Eq. 18b:

$$n^* \approx \left| \frac{(d_{\mathrm{B.I}} + d_{\mathrm{linker}})\frac{\sin\theta}{\sin\varphi} - d_{\mathrm{C\alpha\beta}}}{l_{\mathrm{c}}} + \left(1 + \frac{\sin\theta}{\sin\varphi}\right) n_{\mathrm{p,MHC}} \right| \ , \tag{20}$$

which can be approximately calculated using contour lengths of length components at force-free state.

By assuming small angle perturbation, which seems reasonable, we further reduce the number of model parameters after relating the tilting angle of the TCR constant domain (φ) and the titling angle of the bonding interface by the following equation:

$$\cot\varphi \approx \frac{d_{\mathrm{N}} - d_{\mathrm{B.I}}}{d_{\mathrm{B.I}} + d_{\mathrm{linker}}}\cot\theta \ , \tag{21}$$

Thus, all terms including φ can be re-expressed by using Eq. 21.

Additionally, the tilting constraint can be expressed as follows:

$$\frac{\tan^{-1}\left(\frac{n_{\mathrm{p,TCR}} l_{\mathrm{c}}}{w_{\mathrm{TCR}}}\right)}{\theta + \varphi} \approx 1 \ , \tag{22}$$

Thus, by using Eq. 19 and 21, only 4 fitting parameters, two structural parameters ($d_{\mathrm{B.I}}$, θ) and two biophysical parameters (k_0, δ_0^*), were used to fit the class II model to data.

C. A general biophysical limit of model parameters

The purpose of this section is to describe a general biophysical limit that constrains the fitted model parameters, which is used in Fig. 6 to accept the correct model application to data of matched MHC class and reject incorrect model application to data of mismatched MHC class. The idea is that, even if the model is capable of fitting experimental data and the parameters are self-consistent with one another within the model, their values should be within known limits. A prototypical example of such a biophysical limit involves the molecular extension per unfolded amino acids. It follows from Eqs. 11 and 17 that the average molecular extension at zero force over all data ($\langle\delta_0\rangle$) should be a linear function of n^* such that $\langle\delta_0\rangle = an^* + b$. The y-axis intercept b can be determined from the slip bond data because slip bonds are not expected to have unfolded amino acids (i.e., $n^* = 0$) but still have a nonzero extension. The slope a is constrained by the fact that the contour length of a single amino acid has a small range (~0.4 ± 0.02 nm/a.a.)[7,13,14]; thus, the average molecular extension ($\langle\delta_0\rangle$) per unfolded amino acid should be bounded by:

$$0 < a < 0.4\,, \qquad \textbf{(23)}$$

Imposing this range limit of $\langle\delta_0\rangle$/a.a. would enable us to rule out inappropriate application of the model even if such application could achieve reasonable level of goodness-of-fit. Indeed, all results were below the biophysical limitation.

Conversely, a nearly zero estimate of a would indicate that the model is inappropriate for catch-slip bond data because, for the model to fit catch-slip bonds, it requires $n^* > 0$ (see Figs. 2c-g and 3e, 3rd row). A parameter estimation of $a \approx 0$ indicates the lack of dependence of the model behavior on n^*, which abolishes the model's ability to predict TCR signaling and antigen discrimination, making the model irrelevant to biology.

The sturdier class II than class I pMHC structure also precludes large rotation during conformational change at transition state, leaving only unfolding and stretching along the force.

Thus, the average molecular extension per amino acid should be close to 0.4 nm/a.a. On the other hand, $a \approx 0$ is expected from fitting the slip-only data because such data correspond to the $n^* = 0$ case, which makes it difficult to robustly estimate the correct a value. Thus, the average molecular extension per amino acid should be well-correlated with each other (*i.e.*, high level of goodness-of-fit as measured by R^2) and in the range between 0 to 0.4 nm/a.a. In Fig. 6, we used these criteria to test the appropriateness of cross-applying the class I model to class II data and *vice versa*, showing that it is appropriate to use either model to fit matched data but inappropriate to use either model to fit mismatch data.

Supplementary Figures

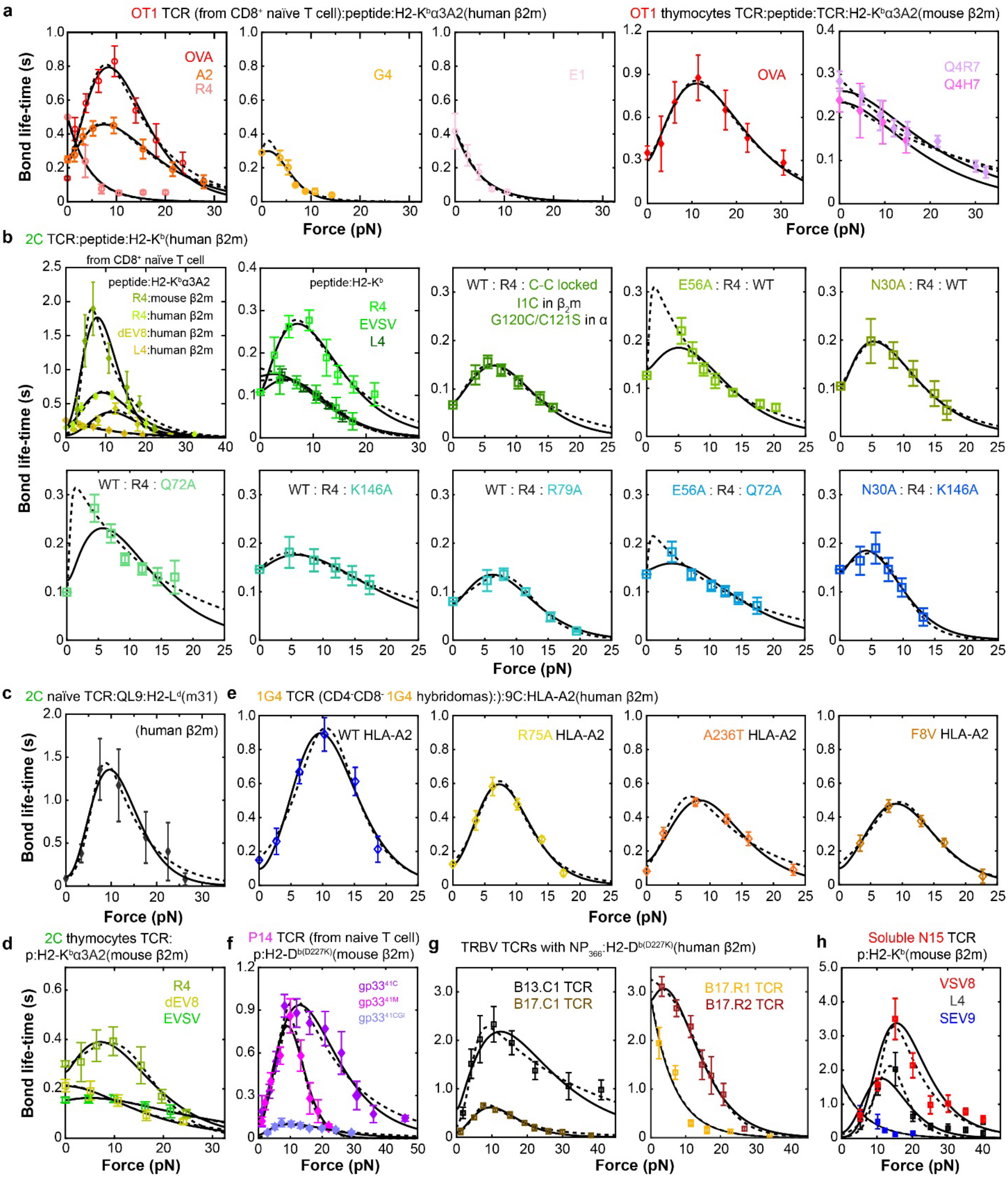

Supplementary Figure 1 | Fitting class I model and two-pathway model to data of TCR–pMHC-I bond lifetime *vs* force. Fitting of theoretical $1/k(F)$ curves of the class I model (solid curves) and the two-pathway model (dashed curves) to experimental bond lifetime *vs* force

data (*points*, Mean ± SEM from at least n > 20 bond lifetime data per each force bin) of 9 TCRs and their mutants interacting with different pMHCs as described below (re-analyzed from Refs. [4,15-19]). **a** OT1 TCR expressed on $CD8^+$ naïve T cells (first three panels) or $CD4^+CD8^+$ thymocytes (last two panels) interacting with indicated p:H2-K^bα3A2. **b** 2C TCR expressed on $CD8^+$ naïve T cells interacting with indicated p:H2-K^bα3A2 (*top*, 1st panel) or on $CD8^-$ hybridomas interacting with indicated peptides presented by WT (*top*, 2nd panel) or disulfate-locked MT (*top*, 3rd panel) H2-K^b, 2C TCR with indicated point mutations expressed on $CD8^-$ hybridomas interacting with R4:H2-K^b (*top*, 4th and 5th panels), 2C TCR expressed on $CD8^-$ hybridomas interacting with R4 peptide presented by H2-K^b with indicated point mutations (*bottom*, 1st-3rd panels), or 2C TCR with indicated point mutations expressed on $CD8^-$ hybridomas interacting with R4 peptide presented by H2-K^b with indicated point mutations (*bottom*, 4th and 5th panels). **c** 2C TCR expressed on $CD8^+$ naïve T cells interacting with indicated p:H2-L^d(m31) with truncated α3 domain. **d** 2C TCR expressed on $CD4^+CD8^+$ thymocytes interacting with indicated p:H2-K^bα3A2. **e** 1G4 TCR expressed on $CD8^-$ hybridomas interacting with indicated p:HLA-A2. **f** P14 TCR expressed on $CD8^+$ naïve T cells interacting with indicated p:H2-D^{bD227K}. **g** TRBV TCRs of canonical (B13.C1 and B17.C1) and reverse (B17.R1 and B17.R2) pMHC-I docking orientation expressed on hybridomas interacting with NP_{366}:H2-D^{bD227K}. **h** Purified N15 TCRαβ interacting with indicated p:H2-K^b. The first panel of **a** and the first two top panels of **b** are replotted from **Fig. 3a** and **b** for completeness. See Supplementary Table 3 for a list of the interacting molecules. Source data are provided as a Source Data file.

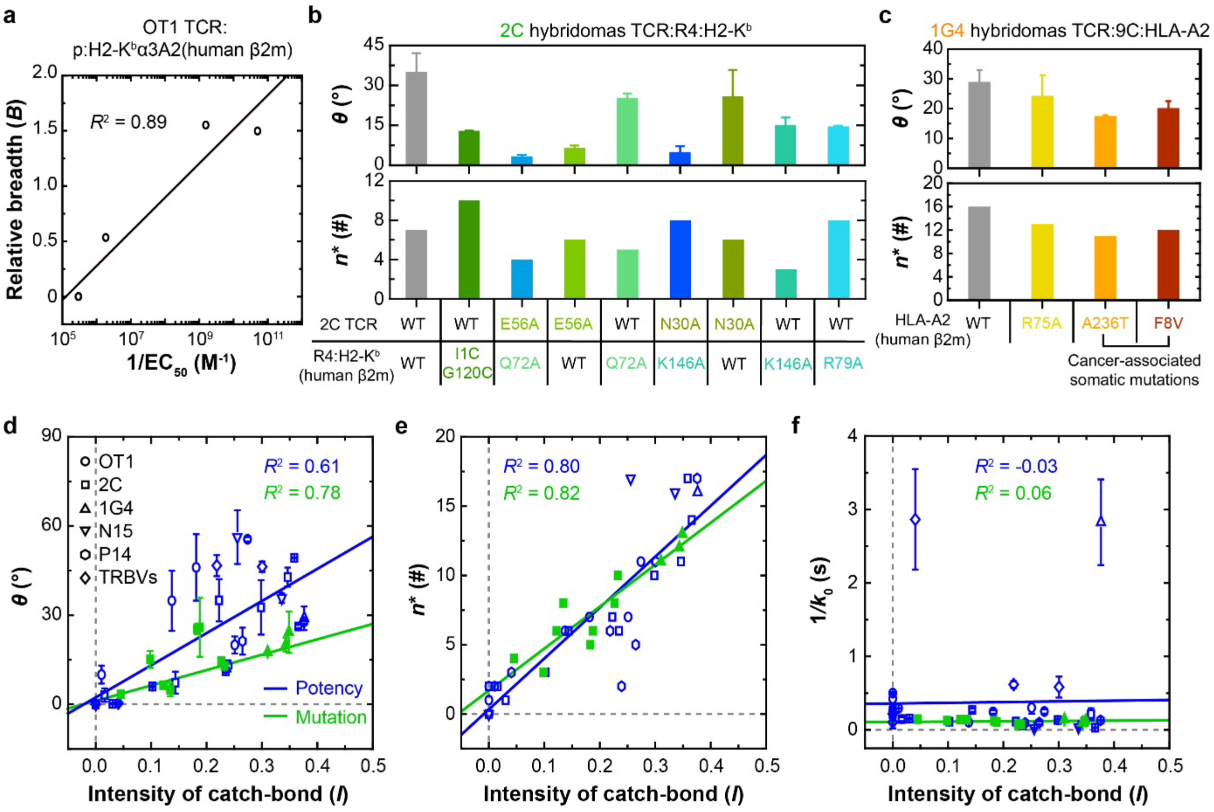

Supplementary Figure 2 | Correlation of model parameters with pMHC-I biological activity and TCR bond type. a Relative breadth *vs* the reciprocal peptide dose required to stimulate half maximal of OT1 T cell proliferation. **b-c** Model parameters $\boldsymbol{\theta}$ (the tilted angle of the bonding interface, *upper*) and $\boldsymbol{n}^*$ (the number of unfolded amino acids, *lower*) that best-fit the data in Supplementary **Fig. 1b** and **d** are plotted *vs* the indicated WT and mutant 2C (**b**) and 1G4 (**c**) TCRs with their indicated pMHCs. **d-f** Scattergrams of $\boldsymbol{\theta}$ (**d**), $\boldsymbol{n}^*$ (**e**), and $\mathbf{1}/\boldsymbol{k_0}$ (**f**) *vs* $\boldsymbol{I} = \boldsymbol{L}/(\mathbf{1} + \boldsymbol{B})$ (catch bond intensity) are plotted using the data from **Figs. 3e**, **4b**, and **Supplementary Figs. 2b** & **2c** to examine correlation. Blue-open symbols indicate data of known T cell biological activities (ligand potencies) induced by the corresponding TCR–pMHC-I interactions that correlate to catch bond intensity. Green-closed symbols indicate data of known effects on catch bond metrics by targeted mutations on the TCR, MHC, or both. All error bars present SE derived from fitting of the model to Mean ± SEM of bond lifetimes (Supplementary Table 3). Source data are provided as a Source Data file.

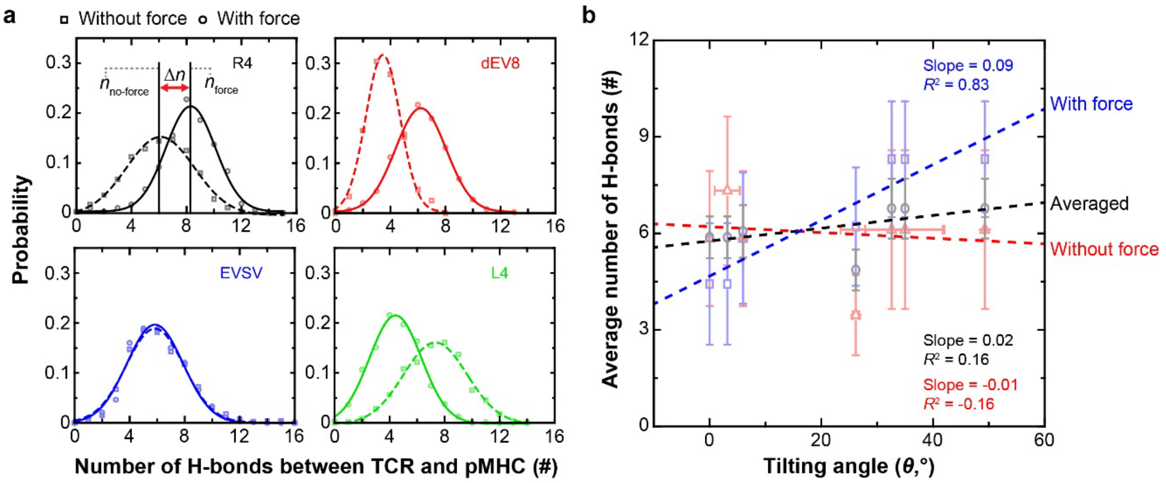

Supplementary Figure 3 | Correlation of force-induced TCR–pMHC-I hydrogen (H) bonds with bonding interface tilting angle. a H-bond distributions at bonding interface between 2C TCR and the indicated pMHCs (R4, dEV8, EVSV, or L4) in the presence (*solid curves*) or absence (*dotted curves*) of the force (obtained by re-analysis of SMD simulation results from Ref. [4] and fitted by Gaussian functions). **b** Plots of average number of H-bonds (Mean ± SD from (**a**)) with respect to fitted tilting angle (with SE from fitting) of bonding interface in the absence (*red*) and presence (*blue*) of force as well as their average (*gray*). Average numbers of H-bonds were determined as mean value for each Gaussian distribution at each titling angle. All colored dashed lines are linear fits. Source data are provided as a Source Data file.

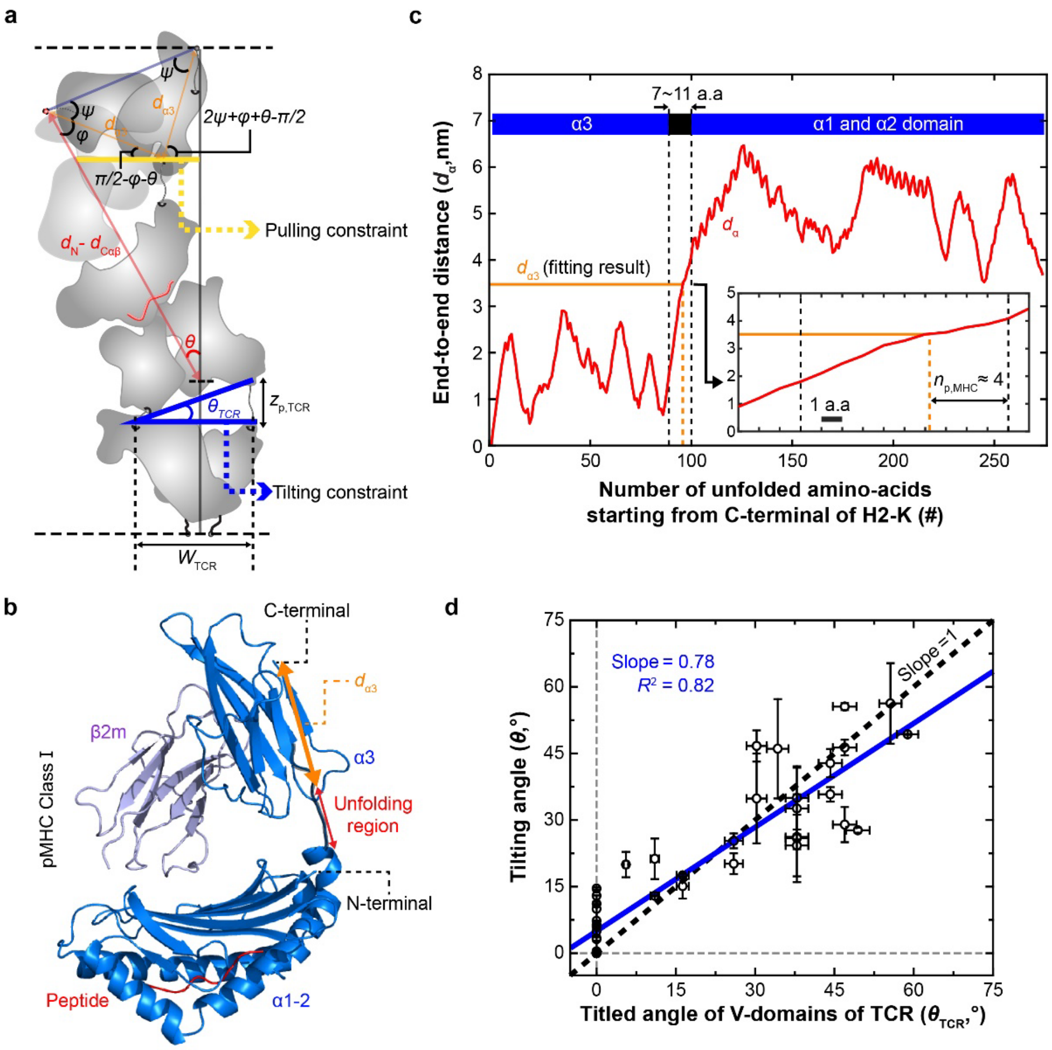

Supplementary Figure 4 | Pulling and tilting constraints of class I model. a An illustration of both pulling and tilting constraints. Length of yellow line was used to set up the equation of the pulling constraint between $\boldsymbol{d_{\alpha 3}}$ and $\boldsymbol{\theta}$. The tilted angle from the blue lines was used to check asymmetric stretching of TCR. **b** A pMHC-I structure based on PDB code 2CKB. Partial unfolding of MHC is assumed to start from the end of the α1-α2 domain towards the α3 domain as suggested by SMD simulation[4]. **c** Representative end-to-end distance of $\boldsymbol{d_{\alpha 3}}$ *vs* the number of unfolded amino acids. The distance is calculated from C-terminal end of the **α3** domain based on the structure shown in (**b**). Distance is calculated using the PBD structure for each construct. $\boldsymbol{d_{\alpha 3}}$ distance obtained from fitting the model to data is converted to $\boldsymbol{n_{p,MHC}}$ (*inset*). **d** Tilted angle of the TCR variable domains ($\boldsymbol{\theta_{TCR}}$) *vs* the titled angle of bonding interface ($\boldsymbol{\theta}$)

as one of the fitting parameters. The validity of asymmetric stretching of TCR was checked by the linear relationship between the two angles. All error bars present SE derived from fitting of the model to Mean ± SEM of bond lifetimes. Source data are provided as a Source Data file.

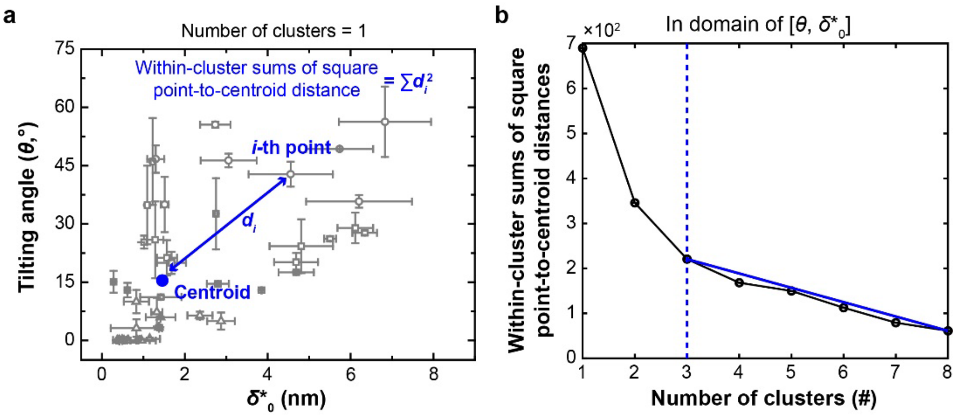

Supplementary Figure 5 | Determining the number of clusters in parameter space. a Definitions of the *i*-th point-to-centroid Euclidean distance (blue line) and of the within-cluster sums of square point-to-centroid distance when the number of clusters is 1. All error bars present SE derived from fitting of the model to Mean ± SEM of bond lifetimes. **b** Sum of all squared Euclidean distances *vs* number of possible clusters. Point of abrupt reduction of steepness of slope leading to a straight line (*blue solid-line*) was identified a number of clusters (*blue dotted-line*). Source data are provided as a Source Data file.

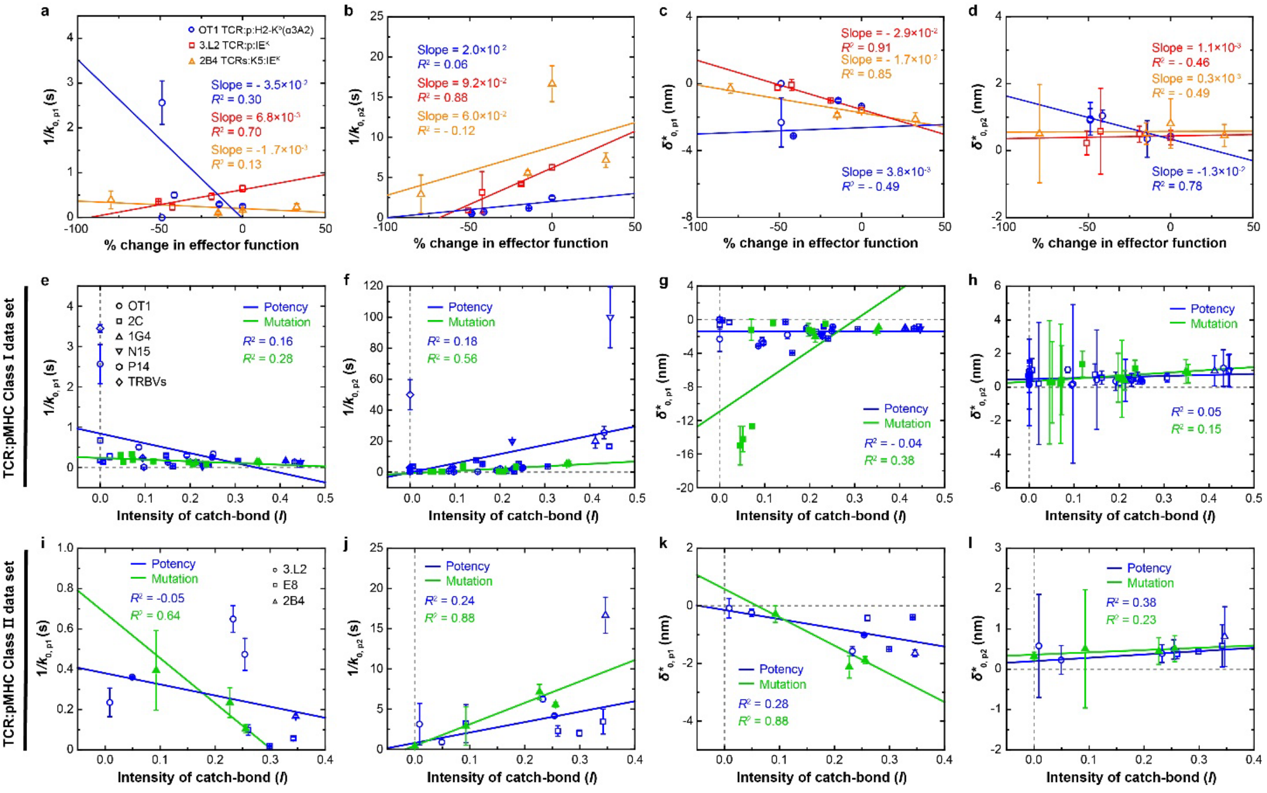

Supplementary Figure 6 | Lack of correlations between best-fit parameters of the two-pathway model and T cell function. a-d Correlation plots of $\mathbf{1/k_{0,p1}}$ (**a**), $\mathbf{1/k_{0,p2}}$ (**b**), $\boldsymbol{\delta^*_{0,p1}}$ (**c**), and $\boldsymbol{\delta^*_{0,p2}}$ (**d**) *vs* % changes in effector function. T cell effector function was defined by the peptide dose required to achieve half-maximal proliferation ($1/EC_{50}$) of OT1 T cells (*blue*) or to generate 40% B cell apoptosis ($1/EC_{40}$)[20] for 3.L2 T cells (*red*), or the area under the dose response curve (AUC) of 2B4 hybridoma IL-2 production[21] (*orange*). Color-matched solid lines are linear fits for the indicated TCR–pMHC systems. Also indicated are the slopes and R^2 values. **e-l** Correlation plots of $\mathbf{1/k_{0,p1}}$ (**e, i**) $\mathbf{1/k_{0,p2}}$ (**f, j**), $\boldsymbol{\delta^*_{0,p1}}$ (**g, k**), and $\boldsymbol{\delta^*_{0,p2}}$ (**h, l**) *vs* the intensity of catch bond evaluated from the indicated class I (**e-h**) or class II (**i-l**) datasets. Solid lines are linear fits, which are color-matched to indicate the 'potency-' or 'mutation-' related datasets for the class I system. All error bars of points present SE derived from fitting of the model to Mean ± SEM of bond lifetimes (Supplementary Table 4). Source data are provided as a Source Data file.

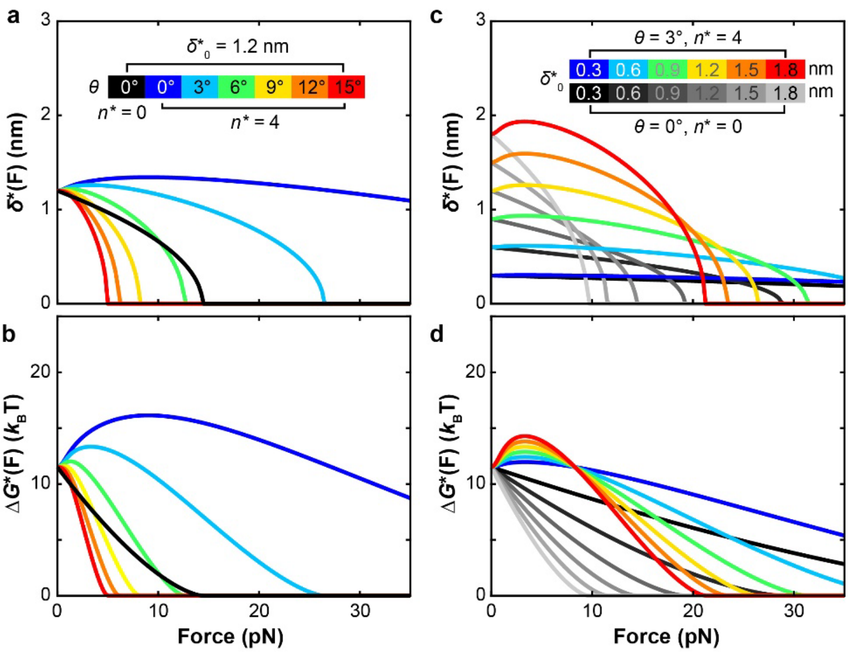

Supplementary Figure 7 | Characterization of the energy landscape of TCR–pMHC-II dissociation. a-d Plots of transition state location $\boldsymbol{\delta}^*$ (**a**, **c**) and height of energy barrier $\Delta \boldsymbol{G}^*$ (**b**, **d**) *vs* force $\boldsymbol{F}$ for changing $\boldsymbol{\theta}$ and $\boldsymbol{n}^*$ (**a**, **b**) or $\boldsymbol{\delta}_0^*$ and $\boldsymbol{n}^*$ (**c**, **d**) while keeping $\boldsymbol{\delta}_0^*$ = 1.2 nm (**a**, **b**) or $\boldsymbol{\theta}$ = 0, 3° (**c**, **d**). Inset color bars indicate parameter values used to plot the color-match theoretical curves. Source data are provided as a Source Data file.

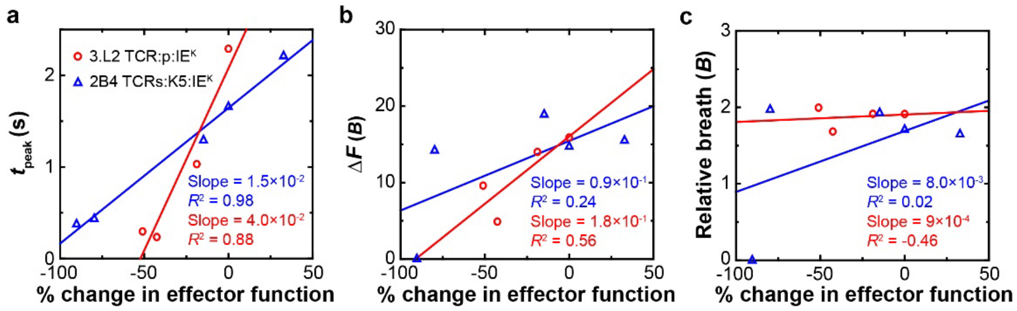

Supplementary Figure 8 | Correlation of metrics of TCR–pMHC-II bond lifetime *vs* force curves with T cell biological activity. a-c Dimensional metrices, $\boldsymbol{t_{\text{peak}}}$ (**a**), $\Delta \boldsymbol{F}$ (**b**) and relative breadth of catch-slip bond $\boldsymbol{B}$ (**c**) *vs* reciprocal % change (relative to WT) in T cell effector function, i.e., the peptide dose required for 3.L2 T cells to generate 40% B cell apoptosis ($1/EC_{40}$)[20] (*red*) or the area under the dose response curve (AUC) of 2B4 hybridoma IL-2 production[21] (*blue*) plots. Source data are provided as a Source Data file.

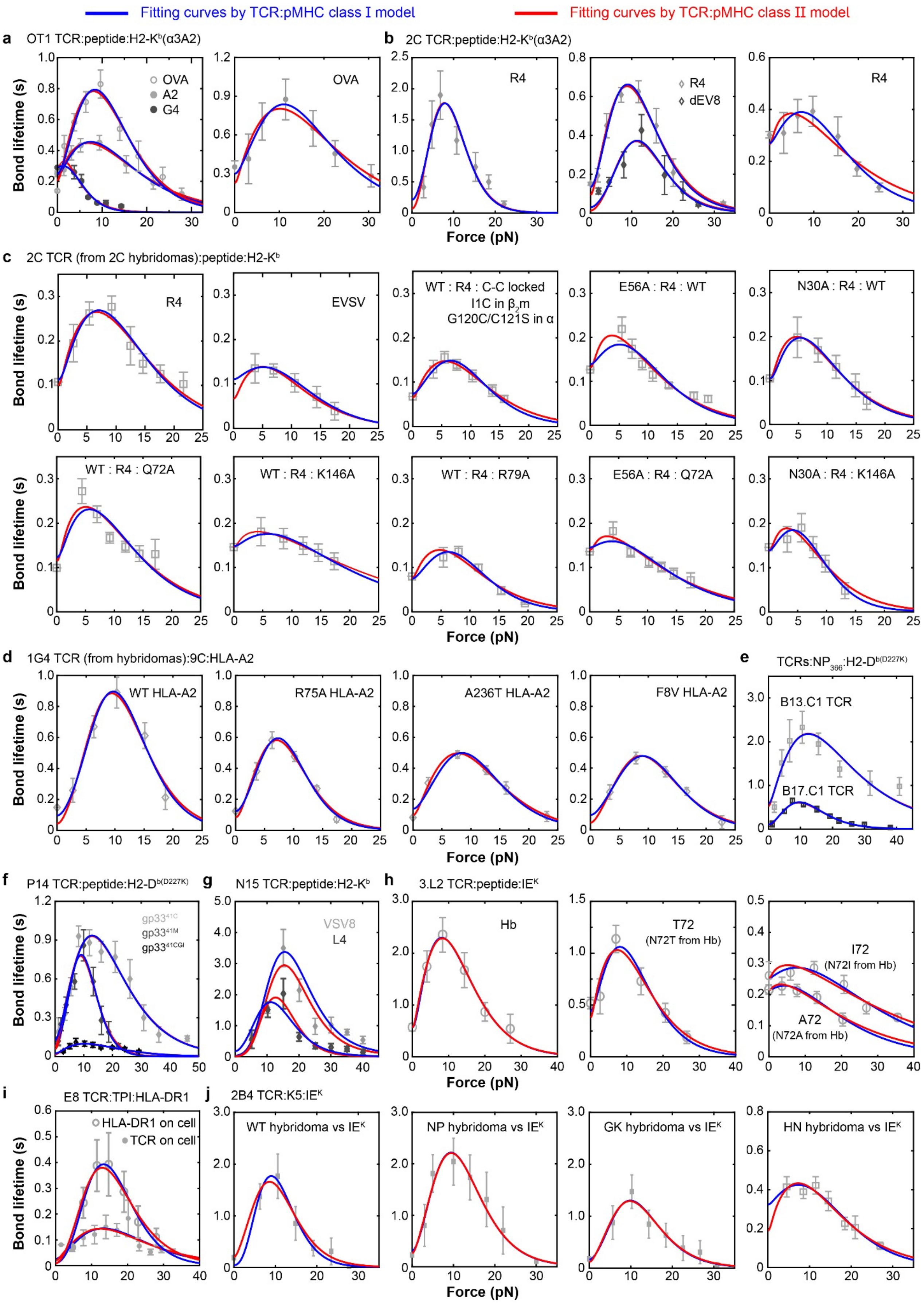

Fitting curves by TCR:pMHC class I model
Fitting curves by TCR:pMHC class II model
a OT1 TCR:peptide:H2-Kb(α3A2)
OVA
A2
G4
b 2C TCR:peptide:H2-Kb(α3A2)
R4
dEV8
c 2C TCR (from 2C hybridomas):peptide:H2-Kb
EVSV
WT : R4 : C-C locked
I1C in β2m
G120C/C121S in α
E56A : R4 : WT
N30A : R4 : WT
WT : R4 : Q72A
WT : R4 : K146A
WT : R4 : R79A
E56A : R4 : Q72A
N30A : R4 : K146A
d 1G4 TCR (from hybridomas):9C:HLA-A2
WT HLA-A2
R75A HLA-A2
A236T HLA-A2
F8V HLA-A2
e TCRs:NP366:H2-Db(D227K)
B13.C1 TCR
B17.C1 TCR
f P14 TCR:peptide:H2-Db(D227K)
gp33 41C
gp33 41M
gp33 41CGI
g N15 TCR:peptide:H2-Kb
VSV8
L4
h 3.L2 TCR:peptide:IEK
Hb
T72
(N72T from Hb)
I72
(N72I from Hb)
A72
(N72A from Hb)
i E8 TCR:TPI:HLA-DR1
HLA-DR1 on cell
TCR on cell
j 2B4 TCR:K5:IEK
WT hybridoma vs IEK
NP hybridoma vs IEK
GK hybridoma vs IEK
HN hybridoma vs IEK
Bond lifetime (s)
Force (pN)

Supplementary Figure 9 | Comparing fitting curves of class I and class II models to the same data. a-j Fitting of theoretical $1/k(F)$ curves predicted by the class I (blue) or class II (*red*) model to experimental lifetime (*points*, Mean ± SEM from at least $n > 20$ bond lifetime data per each force bin) *vs* force data of TCR bonds with pMHC-I (**a-g**) or pMHC-II (**h-j**) ligands, as described below (partly presented in Supplementary Fig. 1, re-analyzed from Refs. [4,15-19,22-24]): OT1 (**a**) or 2C (**b**) TCR expressed on either $CD8^+$ naïve T cells or $CD4^+CD8^+$ thymocytes interacting with indicated p:H2-K^bα3A2; WT or mutant 2C (**c**) or 1G4 (**d**) TCR expressed on hybridomas interacting with indicated peptides presented by WT or MT H2-K^b or HLA-A2. B13.C1 and B17.C1 TCR expressed on hybridomas interacted with NP_{366} bound to the D227K MT of H-$2D^b$ to prevent CD8 binding (**e**). P14 TCR expressed on $CD8^+$ naïve T cells interacting with indicated the D227K MT of H-$2D^b$ to prevent CD8 binding (**f**). Soluble N15 TCRαβ interacting with indicated p:H2-K^b (**g**). 3.L2 TCR expressed on $CD4^-CD8^+$ T cells interacting with indicated p:I-E^k (**h**). E8 TCR expressed on $CD4^-$ Jurkat cells interacting with TPI:HLA-DR1 or TPI:HLA-DR1 expressed on THP-1 cells interacting with E8 TCR (**i**). WT or MT 2B4 TCRs expressed on $CD4^-$ hybridomas interacting with K5:I-E^k (**j**). See Supplementary Table 3 and 6 for lists of the interacting molecules. Source data are provided as a Source Data file.

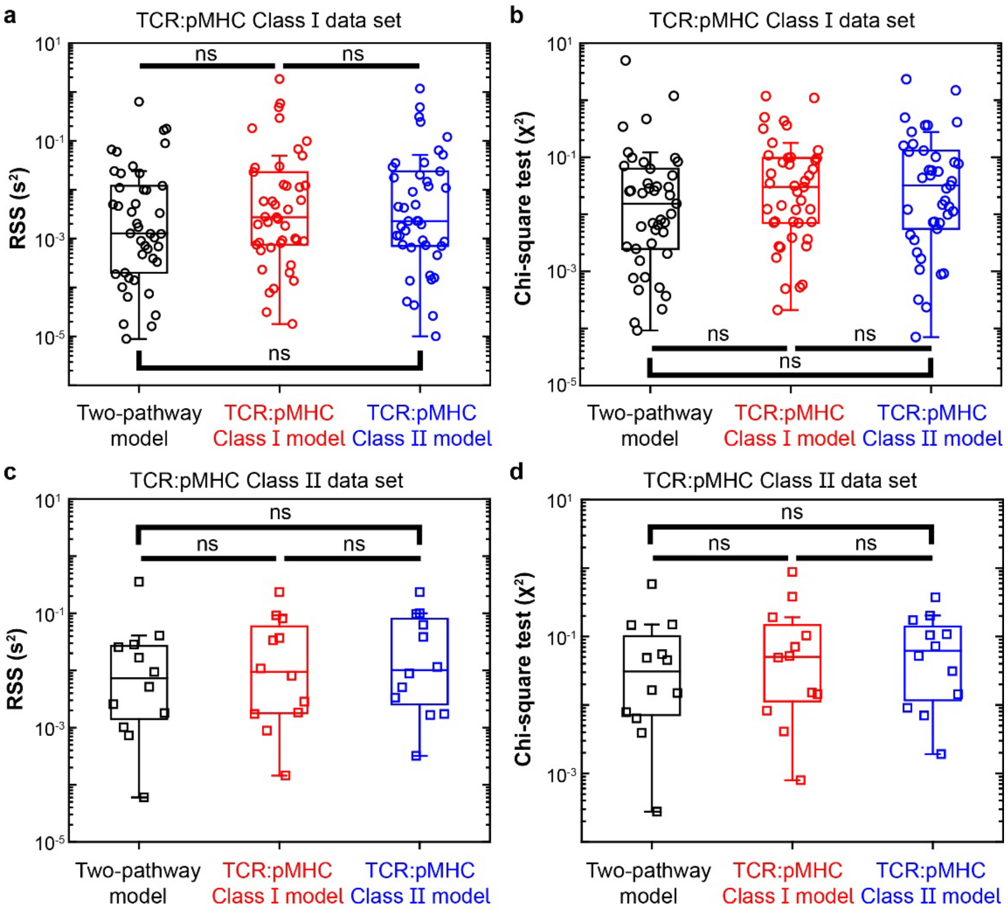

Supplementary Figure 10 | Comparison of goodness-of-fit measures among the class I and II models and the two-pathway model to class I and class II data. a-b Residual sum of squares (RSS, **a**) and Chi-square values (χ^2, **b**) obtained using three models – two-pathway model (*black*), TCR–pMHC-I model (red), and TCR–pMHC-II model (*blue*) – to fit pMHC-I data. **c-d** Residual sum of squares (RSS, **c**) and Chi-square values (χ^2, **d**) obtained using three models – two-pathway model (*black*), TCR-pMHC-I model (red), and TCR-pMHC-II model (*blue*) – to fit pMHC-II data. Individual scattered points, median, 25%, 75% and 5%, 95% whiskers are shown. Statistical tests were done using two-sided paired *t* test. Source data are provided as a Source Data file.

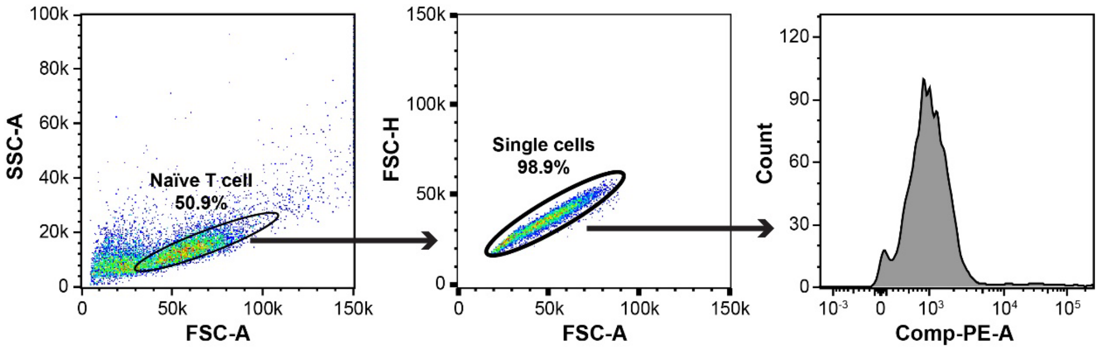

Supplementary Figure 11 | Examples of gating strategy for flow cytometry. Naïve T cells expressing 2C TCR and CD8 from 2C transgenic mice were stained with PE-anti-CD69 and PE-cy7-anti-CD25. Samples were first gated on FSC-A vs SSC-A followed by a secondary gate for single cells based on FSC-A vs FSC-H.

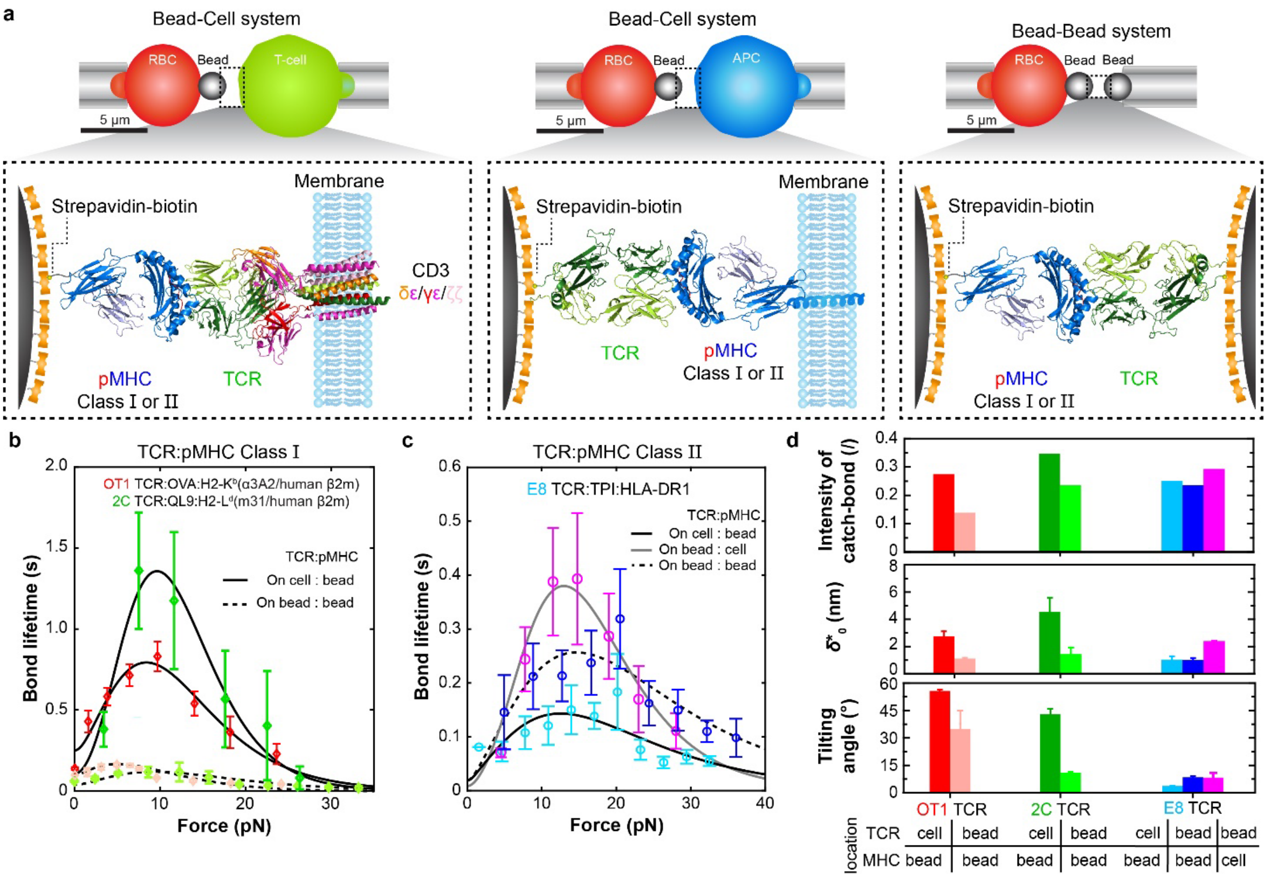

Supplementary Figure 12 | Comparison of pMHC interactions with cell surface and soluble TCRs. a Schematics of three biomembrane force probe (BFP) experiments. The BFP were set up in three configurations (*upper row*) depending on the molecular systems tested (*lower row*). In the first configuration (*left column*), the pMHC was coated on a BFP bead (*left*) and the TCR-CD3 complex was expressed on a T cell (*right*). In the second configuration (*middle column*), the purified TCRαβ was coated on a BFP bead (*left*) and the pMHC was expressed on an APC (*right*). In the third configuration, the pMHC was coated on a BFP bead (*left*) and the purified TCRαβ was coated on a glass bead (*right*). The TCR–pMHC complexes were drawn based on the cryoEM structure with CD3 (6JXR) or crystal structure without CD3 (2CKB). **b-c** Fitting of theoretical $1/k(F)$ (*curves*) to experimental bond lifetime *vs* force data (*points*, Mean ± SEM from n > 50 bond lifetime data per each force bin) of the following interactions: OT1 or 2C TCR respectively expressed on $CD8^+$ naïve T cells (*red*) or $CD8^-$

hybridomas (*green*) or coated on beads (*pink* for OT1 and *yellow-green* for 2C) respectively interacting with OVA:H2-$K^b\alpha$3A2 or QL9:H2-L^d(m3), both MHC class I molecules[22] (**b**) or of E8 TCR expressed on $CD8^-$ Jurkat (*sky-blue*) or coated on beads interacting with TPI:HLA-DR1, a MHC class II, coated on beads (*blue*) or expressed on THP-1 cells (*purple*)[24] (**c**). **d** Comparison of the catch bond intensity (*top*) and best-fit parameters $\boldsymbol{\delta_0^*}$ (*middle*) and $\boldsymbol{\theta}$ (*bottom*) evaluated from data in **b** and **c** of TCR–pMHC interactions measured using the three configurations depicted in a. All error bars present SE derived from fitting of the model to Mean ± SEM of bond lifetimes. Source data are provided as a Source Data file.

Supplementary Table 1 | Summary of model constants

**Parameters**	**Symbol**	**Value**	**Reference**
Elastic modulus of the folded globular domain	E_d	100 pN	[2]
Average contour length	l_c	0.36 nm	[6-8]
Persistence length	l_p	0.39 nm	[6-8]
Elastic modulus of polypeptides	E_p	50 μN	[8]

**TCR : peptide : MHC**	$\boldsymbol{d_{N,c}}$ **(nm)**⁋	$\boldsymbol{d_{C\alpha\beta,c}}$ **(nm)**‡	$\boldsymbol{\varphi}$ **(°)**‖
OT1:peptide:H2-K^{b}	11.6	3.4	23.6
2C:peptide:H2-K^{b}	12.7	3.7	13.8
2C:peptide:H2-L^{d}(m3)	10.9	3.7	5
1G4:peptide:HLA-A2	12.3	3.7	13.3
P14:peptide:H2-D^{b}	12.7	3.4	15
B13.C1, B17.C1, B17.R1, B17.R2:peptide:H2-D^{b}	12.5	3.4	15
N15:peptide:H2-K^{b}	11.6	3.4	23.6
3.L2:peptode:I-E^{k}	12.3	3.4	N.A.
E8:peptide:HLA-DR1	12.3	3.4	N.A.
2B4:peptide:I-E^{k}	12.3	3.4	N.A.

⁋ Force-free extension of bound state

‡ Force-free length of TCRαβ constant domain

‖ Angle shown in Fig. 2b and Supplementary Figure 4

N.A. indicates not applicable.

Supplementary Table 2 | An example of finding the best-fit parameters for OT1 TCR–OVA:H2-K^{b}a3A2 bond

n^*	$d_{\alpha 3}$ (nm)‡	θ (°)‡	$n^* l_c$ (nm)	δ_l (nm)	δ_0 (nm)‡	k_0 (s^{-1})‡
1	5.2	61.9	0.36	-0.8	0.7	4.84
2	5.1	59.7	0.72	-0.5	1.1	4.66
3	4.9	57.3	1.08	0.0	1.4	4.54
4	4.7	55.4	1.44	0.2	1.7	4.44
5	4.5	54.1	1.80	0.6	1.9	4.34
6	4.4	53.5	2.16	1.0	2.1	4.26
7	4.3	53.4	2.52	1.3	2.2	4.20
8	4.2	53.6	2.88	1.7	2.4	4.14
9	4.1	54.1	3.24	2.0	2.5	4.09
10	4.1	54.8	3.60	2.4	2.6	4.04
**11**	**4.0**	**55.5**	**3.96**	**2.7**	**2.7**	**4.00**
12	4.0	56.4	4.32	3.1	2.8	3.73
13	1.8	25.8	4.68	3.5	3.9	3.76
14	2.4	70.5	5.04	3.9	3.3	3.87

‡The fitting parameters derived from model (see Supplementary Model Derivation, Section A.3).

Supplementary Table 3 | TCR–pMHC-I bond summary and their best-fitting parameters

TCR	peptide	MHC class Ⅰ	n^{*}	$d_{\alpha 3}$ ± error (nm)	θ ± error (°)	δ_0 ± error (nm)	k_0 ± error (s^{-1})	$\Delta G_0^{\ddagger}$ ± error (k_BT)
OT1[†]	OVA	H2-K^{b} (α3A2/hβ2m)	11	4.0 ± 0.2	55.5 ± 0.8	2.7 ± 0.4	4.0 ± 0.5	12.4 ± 0.1
OT1[⁋]	OVA	H2-K^{b} (α3A2/hβ2m)	6	4.0 ± 0.5	34.9 ± 10.1	1.1 ± 0.1	9.6 ± 1.5	11.6 ± 0.1
OT1[†]	A2	H2-K^{b} (α3A2/hβ2m)	7	4.0 ± 0.7	46.1 ± 11.2	1.2 ± 0.1	4.1 ± 0.5	12.4 ± 0.1
OT1[†]	G4	H2-K^{b} (α3A2/hβ2m)	2	0.4 ± 0.2	10.0 ± 3.2	0.8 ± 0.3	3.4 ± 0.4	12.6 ± 0.1
OT1[†]	E1	H2-K^{b} (α3A2/hβ2m)	0	N.A.	N.A.	0.9 ± 0.1	2.4 ± 0.6	13.0 ± 0.3
OT1[†]	R4	H2-K^{b} (α3A2/hβ2m)	0	N.A.	N.A.	1.0 ± 0.3	2.0 ± 0.1	13.1 ± 0.1
OT1[*]	OVA	H2-K^{b} (α3A2/mβ2m)	7	1.7 ± 0.3	20.0 ± 2.9	1.7 ± 0.4	3.3 ± 1.2	12.6 ± 0.3
OT1[*]	Q4H7	H2-K^{b} (α3A2/mβ2m)	2	N.A.	N.A.	0.4 ± 0.2	4.3 ± 0.8	12.4 ± 0.2
OT1[*]	Q4R7	H2-K^{b} (α3A2/mβ2m)	1	N.A.	N.A.	0.4 ± 0.1	3.8 ± 0.4	12.5 ± 0.1
2C[†]	R4	H2-K^{b} (α3A2/mβ2m)	17	4.2 ± 0.1	49.3 ± 0.1	5.7 ± 0.8	4.7 ± 2.9	12.3 ± 0.5
2C[†]	R4	H2-K^{b} (α3A2/hβ2m)	10	3.5 ± 0.6	32.6 ± 9.2	2.8 ± 0.1	7.5 ± 1.2	11.8 ± 0.2
2C[†]	dEV8	H2-K^{b} (α3A2/hβ2m)	14	2.1 ± 0.1	26.2 ± 0.6	5.5 ± 0.1	34.4 ± 6.3	10.3 ± 0.2
2C[†]	L4	H2-K^{b} (α3A2/hβ2m)	0	N.A.	N.A.	0.4 ± 0.1	3.8 ± 0.3	12.5 ± 0.1
2C[*]	R4	H2-K^{b} (α3A2/mβ2m)	6	0.9 ± 0.1	7.3 ± 3.7	1.3 ± 0.1	3.7 ± 0.4	12.5 ± 0.1
2C[*]	EVSV	H2-K^{b} (α3A2/mβ2m)	1	0.1 ± 0.1	0.2 ± 0.1	0.5 ± 0.1	6.5 ± 0.5	11.9 ± 0.1
2C[*]	dEV8	H2-K^{b} (α3A2/mβ2m)	2	N.A.	N.A.	0.5 ± 0.1	4.7 ± 0.7	12.3 ± 0.1
2C	R4	H2-K^{b} (hβ2m)	7	4.6 ± 0.3	35.0 ± 7.1	1.5 ± 0.1	8.9 ± 7.7	11.6 ± 0.1
2C	R4	H2-K^{b} C-C locked[‡] (hβ2m)	10	1.1 ± 0.1	12.9 ± 0.1	3.8 ± 0.1	13.6 ± 1.1	11.2 ± 0.1
2C	EVSV	H2-K^{b} (hβ2m)	3	0.4 ± 0.2	6.0 ± 0.2	1.4 ± 0.4	9.0 ± 5.3	11.6 ± 0.5
2C	L4	H2-K^{b} (hβ2m)	2	0.2 ± 0.1	3.2 ± 2.3	0.8 ± 0.6	6.9 ± 1.6	11.9 ± 0.2
2C E56A	R4	H2-K^{b} (hβ2m)	6	0.6 ± 0.1	6.4 ± 1.0	2.4 ± 0.3	7.2 ± 0.2	11.8 ± 0.1
2C N30A	R4	H2-K^{b} (hβ2m)	6	4.7 ± 0.3	25.9 ± 9.9	1.3 ± 0.2	9.5 ± 0.5	11.6 ± 0.1
2C	R4	H2-K^{b} Q72A (hβ2m)	5	4.7 ± 0.1	25.3 ± 1.7	1.0 ± 0.1	8.1 ± 0.6	11.7 ± 0.1
2C	R4	H2-K^{b} K146A (hβ2m)	3	4.6 ± 0.4	15.1 ± 2.8	0.3 ± 0.1	8.0 ± 0.2	11.7 ± 0.1
2C	R4	H2-K^{b} R79A (hβ2m)	8	1.0 ± 0.1	14.6 ± 0.3	2.8 ± 0.3	13.3 ± 0.5	11.2 ± 0.1
2C E56A	R4	H2-K^{b} Q72A (hβ2m)	4	0.3 ± 0.1	3.4 ± 0.6	1.4 ± 0.1	6.9 ± 0.2	11.9 ± 0.1
2C N30A	R4	H2-K^{b} K146A (hβ2m)	8	0.8 ± 0.2	5.0 ± 2.2	2.9 ± 0.3	7.2 ± 0.2	11.8 ± 0.1
2C[†]	QL9	H2-L^{d} (m31/hβ2m)	11	3.7 ± 0.2	42.8 ± 3.2	4.6 ± 1.0	8.9 ± 6.0	11.6 ± 0.5
2C[⁋]	QL9	H2-L^{d} (m31/hβ2m)	6	1.7 ± 0.3	11.2 ± 0.4	1.4 ± 0.5	17.6 ± 5.8	10.8 ± 0.1
1G4	9C	HLA-A2 (hβ2m)	16	2.2 ± 0.2	29.0 ± 4.0	6.1 ± 0.4	10.3 ± 2.6	11.5 ± 0.3

1G4	9C	HLA-A2 R75A (hβ2m)	13	2.4 ± 0.5	24.3 ± 6.9	4.8 ± 0.8	8.1 ± 0.3	11.7 ± 0.1
1G4	9C	HLA-A2 A236T (hβ2m)	11	1.3 ± 0.1	17.5 ± 0.3	4.7 ± 0.4	7.2 ± 0.9	11.8 ± 0.1
1G4	9C	HLA-A2 F8V (hβ2m)	12	1.7 ± 0.2	20.2 ± 2.3	4.7 ± 0.5	10.3 ± 2.8	11.5 ± 0.3
P14[†]	Gp33(41C)	H-2D^b (D227K/mβ2m)	5	2.9 ± 0.2	21.3 ± 4.5	1.6 ± 0.2	7.7 ± 0.2	11.8 ± 0.1
P14[†]	Gp33(41M)	H-2D^b (D227K/mβ2m)	17	2.3 ± 0.1	27.7 ± 0.6	6.3 ± 0.3	10.8 ± 1.2	11.4 ± 0.1
P14[†]	Gp33 (41CGI)	H-2D^b (D227K/mβ2m)	2	3.2 ± 0.1	13.0 ± 1.9	0.6 ± 0.1	41.9 ± 3.3	10.1 ± 0.1
N15[¶]	VSV8	H2-K^b (mβ2m)	17	3.3 ± 0.5	56.3 ± 9.1	4.5 ± 0.5	26.8 ± 5.3	10.5 ± 0.2
N15[¶]	L4	H2-K^b (mβ2m)	16	2.1 ± 0.1	35.8 ± 1.6	4.3 ± 0.5	5.6 ± 41.7	12.1 ± 0.9
N15[¶]	SEV9	H2-K^b (mβ2m)	0	N.A.	N.A.	0.6 ± 0.1	1.6 ± 0.1	14.3 ± 0.2
B13.C 1[#]	NP$_{366}$	H-2D^b (D227K/hβ2m)	6	4.9 ± 0.2	46.7 ± 3.5	1.3 ± 0.2	1.7 ± 0.6	13.3 ± 0.3
B17.C 1[#]	NP$_{366}$	H-2D^b (D227K/hβ2m)	11	4.5 ± 0.3	46.3 ± 1.8	3.1 ± 0.7	9.7 ± 3.0	11.5 ± 0.4
B17.R 1[#]	NP$_{366}$	H-2D^b (D227K/hβ2m)	0	N.A.	N.A.	0.6 ± 0.2	0.3 ± 0.1	14.9 ± 0.3
B17.R 2[#]	NP$_{366}$	H-2D^b (D227K/hβ2m)	3	0.3 ± 0.2	0.4 ± 0.3	1.2 ± 0.2	0.4 ± 0.1	14.9 ± 0.2

[⁋]Purified recombinant TCRs coated on beads were used in BFP measurement.
[*]OT1 or 2C TCR expressed on $CD4^+CD8^+$ thymocytes were used.
[†]OT1, P14 or 2C TCR expressed on naïve T cells from transgenic mice were used. In other cases, 2C or 1G4 TCR expressed on $58\alpha^{-1}\beta^{-1}$ hybridomas and J76 Jurkat cells were used.
[¶]Soluble mouse N15 TCRαβ was used to measure bond lifetime in optical tweezers[19].
[#]Mouse TRBV TCRs (B13.C1 and B17.C1 with canonical docking orientation and B17.R1 and B17.R2 with reverse docking orientation) expressed on 5KC hybridomas interacted with NP$_{366}$ bound to the D227K MT of H-2D^b to prevent CD8 binding[18]
[‡]I1C mutation in β2m domain, G120C and C121S mutations in α domain (H2-K^b) were introduced.
N.A. indicates not applicable.
Note that the best-fit parameters were chosen by the procedure shown in Supplementary Table 2 or determined as the parameter set with the closest δ_1 value to δ_0^* as well as the smallest RSS. All errors of fitting parameters are from the standard errors of bond lifetimes after optimizing the best-fit parameters. All values were rounded to one decimal place.

Supplementary Table 4 | Two-pathway model summary and their best-fitting parameters

TCR-pMHC class I

TCR	peptide	MHC class Ⅰ	$k_{0,p1}$ (s^{-1})	$k_{0,p1}$ error	$\delta^*_{0,p1}$ (nm)	$\delta^*_{0,p1}$ error	$k_{0,p2}$ (s^{-1})	$k_{0,p2}$ error	$\delta^*_{0,p2}$ (nm)	$\delta^*_{0,p2}$ error
OT1[†]	OVA	H2-K^b (α3A2/hβ2m)	3.99	0.49	-1.36	0.05	0.41	0.04	0.43	0.02
OT1[⁋]	OVA	H2-K^b (α3A2/hβ2m)	7.77	0.98	-1.81	0.45	3.29	0.11	0.44	2.96
OT1[†]	A2	H2-K^b (α3A2/hβ2m)	3.36	0.44	-1.01	0.07	0.86	0.04	0.35	0.55
OT1[†]	G4	H2-K^b (α3A2/hβ2m)	1.99	0.26	-3.13	0.04	1.47	0.26	1.04	0.17
OT1[†]	E1	H2-K^b (α3A2/hβ2m)	0.39	0.09	-2.32	1.47	2.01	0.57	0.95	0.49
OT1[†]	R4	H2-K^b (α3A2/hβ2m)	N.A.	N.A.	N.A.	N.A.	2	0.74	0.91	0.35
OT1[*]	OVA	H2-K^b (α3A2/mβ2m)	3.02	0.77	-0.84	0.04	0.35	0.01	0.33	0.03
OT1[*]	Q4H7	H2-K^b (α3A2/mβ2m)	N.A.	N.A.	N.A.	N.A.	4.09	2.54	0.13	1.42
OT1[*]	Q4R7	H2-K^b (α3A2/mβ2m)	N.A.	N.A.	N.A.	N.A.	3.44	1.81	0.16	1.47
2C[†]	R4	H2-K^b (α3A2/mβ2m)	29.9	10.39	-3.94	0.08	0.19	0.02	0.56	0.39
2C[†]	R4	H2-K^b (α3A2/hβ2m)	6.09	0.93	-1.13	0.03	0.28	0.03	0.56	0.25
2C[†]	dEV8	H2-K^b (α3A2/hβ2m)	15.44	2.72	-0.81	0.01	0.06	0.01	0.99	0.93
2C[†]	L4	H2-K^b (α3A2/hβ2m)	N.A.	N.A.	N.A.	N.A.	3.85	2.81	0.36	0.68
2C[*]	R4	H2-K^b (α3A2/mβ2m)	3.37	0.34	-0.26	0.04	0.13	0.01	0.75	0.63
2C[*]	EVSV	H2-K^b (α3A2/mβ2m)	3.69	1.49	-0.33	0.22	2.81	1.05	0.22	3.63
2C[*]	dEV8	H2-K^b (α3A2/mβ2m)	1.49	0.15	-0.65	0.47	3.2	0.34	0.28	2.57
2C	R4	H2-K^b (hβ2m)	8.04	0.89	-1.43	0.28	1.46	0.19	0.4	1.25
2C	R4	H2-K^b C-C locked[‡] (hβ2m)	12.63	0.53	-1.43	0.34	2.48	0.31	0.49	2.3
2C	EVSV	H2-K^b (hβ2m)	7.13	1.82	-0.08	0.08	0.28	0.06	1.02	0.68
2C	L4	H2-K^b (hβ2m)	5.62	1.51	0	0.01	0.49	0.63	0.9	0.22
2C E56A	R4	H2-K^b (hβ2m)	5.27	0.33	-12.7	0.01	2.79	0.32	0.41	2.06
2C N30A	R4	H2-K^b (hβ2m)	7.91	0.34	-1.21	0.74	1.63	0.38	0.56	1.45
2C	R4	H2-K^b Q72A (hβ2m)	7.52	0.09	-14.21	1.51	2.83	0.12	0.29	2.42
2C	R4	H2-K^b K146A (hβ2m)	3.02	0.09	-1.21	1.27	3.83	0.08	0.2	3.55
2C	R4	H2-K^b R79A (hβ2m)	12.37	0.51	-0.48	0.21	0.3	0.34	1.12	0.48
2C E56A	R4	H2-K^b Q72A (hβ2m)	3.2	0.29	-15	2.3	4.26	0.3	0.29	3.67
2C N30A	R4	H2-K^b K146A (hβ2m)	6.76	0.36	-0.39	0.35	0.26	0.34	1.37	0.77
2C[†]	QL9	H2-L^d (m31/hβ2m)	14.16	2.9	-2.24	0.02	0.19	0.01	0.53	0.35
2C[⁋]	QL9	H2-L^d (m31/hβ2m)	15.91	0.42	-0.82	0.14	2.02	1.5	0.47	0.05

1G4	9C	HLA-A2 (hβ2m)	6.25	0.77	-0.98	0.03	0.05	0.01	0.97	0.91
1G4	9C	HLA-A2 R75A (hβ2m)	7.3	0.42	-1.36	0.12	0.19	0.03	0.93	0.71
1G4	9C	HLA-A2 A236T (hβ2m)	8.6	1.19	-2.01	0.67	0.82	0.16	0.4	0.58
1G4	9C	HLA-A2 F8V (hβ2m)	7.72	1.53	-0.92	0.01	0.18	0.07	0.8	0.55
P14†	Gp33(41C)	H-2D^b (D227K/mβ2m)	7.49	3.16	-1.44	0.36	0.45	0.08	0.24	0.14
P14†	Gp33(41M)	H-2D^b (D227K/mβ2m)	7.35	1.23	-1.04	0.03	0.04	0.01	1.13	1.09
P14†	Gp33 (41CGI)	H-2D^b (D227K/mβ2m)	125.55	12.12	-2.77	0.16	6.12	1.21	0.19	4.72
N15¶	VSV8	H2-K^b (mβ2m)	33.35	0.01	-1.8	0.05	0.05	0.01	0.43	0.38
N15¶	L4	H2-K^b (mβ2m)	7.96	3.45	-1.12	0.19	0.01	0.01	0.98	0.97
N15¶	SEV9	H2-K^b (mβ2m)	N.A.	N.A.	N.A.	N.A.	0.64	0.54	0.58	0.48
B13. C1#	NP$_{366}$	H-2D^b (D227K/hβ2m)	4.29	0.26	-2.54	0.43	0.32	0.04	0.14	0.14
B17. C1#	NP$_{366}$	H-2D^b (D227K/hβ2m)	14.64	6.1	-1.93	0.28	0.54	0.02	0.41	0.15
B17. R1#	NP$_{366}$	H-2D^b (D227K/hβ2m)	N.A.	N.A.	N.A.	N.A.	0.35	0.01	0.61	0.26
B17. R2#	NP$_{366}$	H-2D^b (D227K/hβ2m)	0.29	0.01	0	0.01	0.02	0.01	0.78	0.76

TCR-pMHC class II

TCR	peptide	MHC class Ⅱ	$k_{0,p1}$ (s^{-1})	$k_{0,p1}$ error	$\delta^*_{0,p1}$ (nm)	$\delta^*_{0,p1}$ error	$k_{0,p2}$ (s^{-1})	$k_{0,p2}$ error	$\delta^*_{0,p2}$ (nm)	$\delta^*_{0,p2}$ error
3.L2	Hb	I-E^k	1.54	0.18	-1.57	0.16	0.16	0.01	0.39	0.22
3.L2	T72	I-E^k	2.11	0.43	-1.01	0.02	0.24	0.01	0.49	0.24
3.L2	A72	I-E^k	4.25	1.83	-0.08	0.34	0.32	1.54	0.58	1.28
3.L2	I72	I-E^k	2.77	0.02	-0.24	0.13	1.13	0.54	0.23	0.36
E8	TPI	HLA-DR1	17.54	2.53	-0.39	0.02	0.29	0.24	0.58	0.53
E8⁋	TPI	HLA-DR1	10.13	3.59	-0.42	0.09	0.44	0.18	0.38	0.12
E8**	TPI	HLA-DR1	53.09	8.79	-1.5	0.01	0.5	0.12	0.44	0.06
2B4 NP	K5	I-Ek	4.26	1.95	-2.12	0.39	0.14	0.02	0.45	0.33
2B4 WT	K5	I-Ek	5.99	0.17	-1.64	0.12	0.06	0.01	0.81	0.74
2B4 GK	K5	I-Ek	9.53	2.28	-1.88	0.12	0.18	0.01	0.51	0.32
2B4 HN	K5	I-Ek	2.53	2.53	-0.29	0.29	0.35	1.63	0.51	1.47
2B4 NH	K5	I-Ek	N.A.	N.A.	N.A.	N.A.	2.58	2.35	0.32	0.1

⁋Purified recombinant TCRs coated on beads were used in BFP measurement.

*OT1 or 2C TCR expressed on CD4$^+$CD8$^+$ thymocytes were used.

†OT1, P14 or 2C TCR expressed on naïve T cells from transgenic mice were used. In other cases, 2C or 1G4 TCR expressed on 58α^{-1}β^{-1} hybridomas and J76 Jurkat cells were used.

¶Soluble mouse N15 TCRαβ was used to measure bond lifetime in optical tweezers[19].

#Mouse TRBV TCRs (B13.C1 and B17.C1 with canonical docking orientation and B17.R1 and B17.R2 with reverse docking orientation) expressed on 5KC hybridomas interacted with NP$_{366}$ bound to the D227K MT of H-2D^b to prevent CD8 binding[18]

‡I1C mutation in β2m domain, G120C and C121S mutations in α domain (H2-K^b) were introduced.

**The recombinant TCR coated to the BFP bead was tested against pMHC expressed on a THP-1 cell.

N.A. indicates not applicable.

Note that the best-fit parameters were extracted from equation (4) (two-pathway model) in the main text. All errors of fitting parameters are from the standard errors of bond lifetimes after optimizing the best-fit parameters.

Supplementary Table 5 | An example of finding the best-fit parameters for the 3.L2 TCR–Hb:I-E^{k} bond

n^{*}_{MHC} †	$d_{\mathrm{B.I}}$ (nm)‡	θ (°)‡	$n^{*}l_{\mathrm{c}}$ (nm)	δ_{I} (nm)	δ_{0} (nm)‡	k_{0} (s^{-1})‡
1	8.9	0.0	0.4	0.6	0.7	2.0
2	8.8	0.0	0.7	0.9	1.2	1.9
3	8.6	0.5	1.1	1.3	1.5	1.8
**4**	**8.0**	**3.2**	**1.4**	**1.6**	**1.6**	**1.8**
5	7.9	2.6	1.8	2.0	1.7	1.7
6	7.8	2.1	2.2	2.4	1.9	1.7
7	8.0	1.5	2.5	2.7	2.1	1.6
8	7.8	0.6	2.9	3.1	2.2	1.6

† n^{*} can be estimated from n^{*}_{pMHC}.

‡ See Supplementary Model Derivations, Section B.

Supplementary Table 6 | TCR–pMHC-II bond summary and their best-fit parameters

TCR	peptide	MHC class II	n^*	$d_{\mathrm{B.I}}$ ± error (nm)	θ ± error (°)	δ_0 ± error (nm)	k_0 ± error (s^{-1})	$\Delta G_0^{\ddagger}$ ± error (k_BT)
3.L2	Hb	I-E^k	8	8.0 ± 0.2	3.2 ± 1.5	1.6 ± 0.3	1.8 ± 0.2	13.2 ± 0.1
3.L2	T72	I-E^k	6	8.7 ± 0.1	N.A.	1.2 ± 0.1	2.6 ± 0.3	12.9 ± 0.1
3.L2	A72	I-E^k	2	9.3 ± 0.2	N.A.	0.2 ± 0.1	4.7 ± 0.2	12.3 ± 0.1
3.L2	I72	I-E^k	2	9.1 ± 0.1	N.A.	0.1 ± 0.0	3.9 ± 0.3	12.4 ± 0.1
E8	TPI	HLA-DR1	4	8.5 ± 0.1	3.8 ± 0.4	1.0 ± 0.3	50.2 ± 4.8	9.9 ± 0.1
E8⁋	TPI	HLA-DR1	4	7.9 ± 0.1	8.5 ± 0.5	1.1 ± 0.3	51.0 ± 10.5	9.9 ± 0.3
E8*	TPI	HLA-DR1	8	7.6 ± 0.2	8.4 ± 2.4	2.4 ± 0.4	114.0 ± 64	9.1 ± 0.1
2B4 (NP)	K5	I-E^k	14	7.5 ± 0.3	6.7 ± 1.2	2.5 ± 0.2	3.7 ± 0.2	12.5 ± 0.1
2B4 (WT)	K5	I-E^k	16	7.5 ± 0.4	6.1 ± 1.1	2.9 ± 0.4	4.9 ± 6.7	12.2 ± 0.9
2B4 (GK)	K5	I-E^k	10	8.3 ± 0.5	0.3 ± 0.2	2.6 ± 0.3	8.4 ± 1.2	11.7 ± 0.2
2B4 (NH)	K5	I-E^k	0	N.A.	N.A.	0.3 ± 0.1	2.6 ± 0.6	12.9 ± 0.3
2B4 (HN)	K5	I-E^k	4	8.9 ± 0.1	0.3 ± 0.1	0.7 ± 0.3	5.3 ± 0.5	12.2 ± 0.1

⁋The recombinant TCR coated to the bead was tested against pMHC on the BFP bead.
*The recombinant TCR coated to the BFP bead was tested against pMHC expressed on a THP-1 cell.
N.A. indicates not applicable.
Note that the best-fit parameters were chosen using the procedure shown in Supplementary Table 5 or determined as the parameter set with the $\delta_z(n^*)$ value closest to δ_0^* as well as the smallest RSS. All errors of fitting parameters are from the standard errors of bond lifetimes after optimizing the best-fit parameters. All values were rounded to one decimal place.

Supplementary References

1 Smith, S. B., Finzi, L. & Bustamante, C. Direct mechanical measurements of the elasticity of single DNA molecules by using magnetic beads. *Science* **258**, 1122-1126, (1992).

2 Guo, S., Tang, Q., Yao, M., You, H., Le, S., Chen, H. & Yan, J. Structural-elastic determination of the force-dependent transition rate of biomolecules. *Chem Sci* **9**, 5871-5882, (2018).

3 Rossjohn, J., Gras, S., Miles, J. J., Turner, S. J., Godfrey, D. I. & McCluskey, J. T cell antigen receptor recognition of antigen-presenting molecules. *Annual review of immunology* **33**, 169-200, (2015).

4 Wu, P., Zhang, T., Liu, B., Fei, P., Cui, L., Qin, R., Zhu, H., Yao, D., Martinez, R. J., Hu, W., An, C., Zhang, Y., Liu, J., Shi, J., Fan, J., Yin, W., Sun, J., Zhou, C., Zeng, X., Xu, C., Wang, J., Evavold, B. D., Zhu, C., Chen, W. & Lou, J. Mechano-regulation of Peptide-MHC Class I Conformations Determines TCR Antigen Recognition. *Mol Cell* **73**, 1015-1027 e1017, (2019).

5 Bouchiat, C., Wang, M. D., Allemand, J., Strick, T., Block, S. M. & Croquette, V. Estimating the persistence length of a worm-like chain molecule from force-extension measurements. *Biophysical journal* **76**, 409-413, (1999).

6 Seol, Y., Li, J., Nelson, P. C., Perkins, T. T. & Betterton, M. D. Elasticity of short DNA molecules: theory and experiment for contour lengths of 0.6-7 microm. *Biophysical journal* **93**, 4360-4373, (2007).

7 Gebhardt, J. C., Bornschlogl, T. & Rief, M. Full distance-resolved folding energy landscape of one single protein molecule. *Proceedings of the National Academy of Sciences of the United States of America* **107**, 2013-2018, (2010).

8 Choi, H. K., Min, D., Kang, H., Shon, M. J., Rah, S. H., Kim, H. C., Jeong, H., Choi, H. J., Bowie, J. U. & Yoon, T. Y. Watching helical membrane proteins fold reveals a common N-to-C-terminal folding pathway. *Science* **366**, 1150-1156, (2019).

9 Schuler, B., Lipman, E. A. & Eaton, W. A. Probing the free-energy surface for protein folding with single-molecule fluorescence spectroscopy. *Nature* **419**, 743-747, (2002).

10 Yang, W. Y. & Gruebele, M. Folding at the speed limit. *Nature* **423**, 193-197, (2003).

11 Kubelka, J., Hofrichter, J. & Eaton, W. A. The protein folding 'speed limit'. *Curr Opin Struct Biol* **14**, 76-88, (2004).

12 Chung, H. S., Louis, J. M. & Eaton, W. A. Experimental determination of upper bound for transition path times in protein folding from single-molecule photon-by-photon trajectories. *Proceedings of the National Academy of Sciences of the United States of America* **106**, 11837-11844, (2009).

13 Lapidus, L. J., Steinbach, P. J., Eaton, W. A., Szabo, A. & Hofrichter, J. Effects of Chain Stiffness on the Dynamics of Loop Formation in Polypeptides. Appendix: Testing a 1-Dimensional Diffusion Model for Peptide Dynamics. *J. Phys. Chem. B* **106**, 11628-11640, (2002).

14 Guo, S., Efremov, A. K. & Yan, J. Understanding the catch-bond kinetics of biomolecules on a one-dimensional energy landscape. *Communications Chemistry* **2**, 30, (2019).

15 Liu, B., Chen, W., Evavold, B. D. & Zhu, C. Accumulation of dynamic catch bonds between TCR and agonist peptide-MHC triggers T cell signaling. *Cell* **157**, 357-368, (2014).

16 Hong, J., Ge, C., Jothikumar, P., Yuan, Z., Liu, B., Bai, K., Li, K., Rittase, W., Shinzawa, M., Zhang, Y., Palin, A., Love, P., Yu, X., Salaita, K., Evavold, B. D., Singer, A. & Zhu, C. A TCR mechanotransduction signaling loop induces negative selection in the thymus. *Nat Immunol* **19**, 1379-1390, (2018).

17 Kolawole, E. M., Andargachew, R., Liu, B., Jacobs, J. R. & Evavold, B. D. 2D Kinetic Analysis of TCR and CD8 Coreceptor for LCMV GP33 Epitopes. *Front Immunol* **9**, 2348, (2018).

18 Zareie, P., Szeto, C., Farenc, C., Gunasinghe, S. D., Kolawole, E. M., Nguyen, A., Blyth, C., Sng, X. Y. X., Li, J., Jones, C. M., Fulcher, A. J., Jacobs, J. R., Wei, Q., Wojciech, L., Petersen, J., Gascoigne, N. R. J., Evavold, B. D., Gaus, K., Gras, S., Rossjohn, J. & La Gruta, N. L. Canonical T cell receptor docking on peptide-MHC is essential for T cell signaling. *Science* **372**, (2021).

19 Das, D. K., Feng, Y., Mallis, R. J., Li, X., Keskin, D. B., Hussey, R. E., Brady, S. K., Wang, J. H., Wagner, G., Reinherz, E. L. & Lang, M. J. Force-dependent transition in the T-cell receptor beta-subunit allosterically regulates peptide discrimination and pMHC bond lifetime. *Proceedings of the National Academy of Sciences of the United States of America* **112**, 1517-1522, (2015).

20 Kersh, G. J. & Allen, P. M. Structural basis for T cell recognition of altered peptide ligands: a single T cell receptor can productively recognize a large continuum of related ligands. *J Exp Med* **184**, 1259-1268, (1996).

21 Natarajan, A., Nadarajah, V., Felsovalyi, K., Wang, W., Jeyachandran, V. R., Wasson, R. A., Cardozo, T., Bracken, C. & Krogsgaard, M. Structural Model of the Extracellular Assembly of the TCR-CD3 Complex. *Cell Rep* **14**, 2833-2845, (2016).

22 Liu, B., Chen, W., Natarajan, K., Li, Z., Margulies, D. H. & Zhu, C. The cellular environment regulates in situ kinetics of T-cell receptor interaction with peptide major histocompatibility complex. *European journal of immunology* **45**, 2099-2110, (2015).

23 Hong, J., Persaud, S. P., Horvath, S., Allen, P. M., Evavold, B. D. & Zhu, C. Force-regulated In situ TCR-peptide-bound MHC class II kinetics determine functions of CD4+ T cells. *Journal of immunology* **195**, 3557-3564, (2015).

24 Rushdi, M. N., Pan, V., Li, K., Travaglino, S., Choi, H.-K., Hong, J., Griffitts, F., Agnihotri, P., Mariuzza, R. A., Ke, Y. & Zhu, C. Cooperative binding of T cell receptor and CD4 to peptide-MHC enhances antigen sensitivity. *Nature communications* **13**, 7055, (2022).
