# Supplementary material for: Catch bond models may explain how force amplifies TCR signaling and antigen discrimination
Source: Nat Commun. 2023 May 5;14:2616. doi: 10.1038/s41467-023-38267-1 (PMC10163261; doi:10.1038/s41467-023-38267-1)
Supplement: Supplementary file 3 — Description of Additional Supplementary Files [file 41467_2023_38267_MOESM3_ESM.pdf]

Description of Additional Supplementary Files

Supplementary Movie. 1 | SMD simulated trajectories for pulling the 2C TCR–SIYR:H2-K^b complex. The C-terminal residue of H-2K^b was harmonically constrained and the C terminus of TCR was pulled by a dummy spring moving at ~0.1 nm/ns with a spring constant of ~70 pN/nm. The color codes were the same as that in Figure 7a,b and 2C TCRα (blue), 2C TCRβ (red), MHC H-2K^b α (orange), MHC H-2K^b β2m (cyan), and R4 peptide (green). Three different pathways of conformational changes were observed in the pMHC.

Supplementary Movie. 2 | CMD and SMD simulated trajectories side-by-side comparison of pulling effect on the human TCR-CD3 complex focusing on interaction of P223 (TCRβ) and L90 (CD3ε). The left panel shows the freely evolved fluctuations without force from CMD, and the right panel presents the constrained dynamics with force from SMD. For SMD trajectory, the external force of 175pN was exerted on the N-terminal of TCRα, pulling upwards while keeping the C-termini of CD3ε anchored. The color codes were the same as that in Fig. 7g: TCRα (yellow-green), TCRβ (green), and CD3 subunits (golden). The focused interacting residues were highlighted using spheres, where P223β (red) was pulled closer to L90ε (gray) in the presence of force.

Supplementary Movie. 3 | CMD and SMD simulated trajectories side-by-side comparison of pulling effect on the human TCR-CD3 complex focusing on interaction of K183 (TCRβ) and L90 (CD3ε'). The left panel shows the freely evolved fluctuations without force from CMD, and the right panel presents the constrained dynamics with force from SMD. For SMD trajectory, the external force of 175pN was exerted on the N-terminal of TCRα, pulling upwards while keeping the C-termini of CD3ε anchored. The color codes were the same as that in Fig. 7g: TCRα (yellow-green), TCRβ (green), and CD3 subunits (golden). The focused interacting residues were highlighted using spheres, where K183β (blue) was pulled away from L90ε' (gray) in the presence of force.

Supplementary Movie. 4 | CMD and SMD simulated trajectories side-by-side comparison of pulling effect on the human TCR-CD3 complex focusing on interaction of N225 (TCRβ) and E38 (CD3γ). The left panel shows the freely evolved fluctuations without force from CMD, and the right panel presents the constrained dynamics with force from SMD. For SMD trajectory, the external force of 175pN was exerted on the N-terminal of TCRα, pulling upwards while keeping the C-termini of CD3ε anchored. The color codes were the same as that in Fig. 7g: TCRα (yellow-green), TCRβ (green), and CD3 subunits (golden). The focused interacting residues were highlighted using spheres, where N225β (purple) was pulled away from E38γ (gray) in the presence of force.
