# Supplementary material for: Catch bond models may explain how force amplifies TCR signaling and antigen discrimination
Source: Nat Commun. 2023 May 5;14:2616. doi: 10.1038/s41467-023-38267-1 (PMC10163261; doi:10.1038/s41467-023-38267-1)
Supplement: Supplementary file 8 — Reporting Summary [file 41467_2023_38267_MOESM8_ESM.pdf]

nature portfolio

Corresponding author(s): Cheng Zhu

Last updated by author(s): Mar 24, 2023

Reporting Summary

Nature Portfolio wishes to improve the reproducibility of the work that we publish. This form provides structure for consistency and transparency in reporting. For further information on Nature Portfolio policies, see our Editorial Policies and the Editorial Policy Checklist.

Statistics

For all statistical analyses, confirm that the following items are present in the figure legend, table legend, main text, or Methods section.

n/a	Confirmed	
☐	☒	The exact sample size (n) for each experimental group/condition, given as a discrete number and unit of measurement
☐	☒	A statement on whether measurements were taken from distinct samples or whether the same sample was measured repeatedly
☐	☒	The statistical test(s) used AND whether they are one- or two-sided<br>*Only common tests should be described solely by name; describe more complex techniques in the Methods section.*
☐	☒	A description of all covariates tested
☐	☒	A description of any assumptions or corrections, such as tests of normality and adjustment for multiple comparisons
☐	☒	A full description of the statistical parameters including central tendency (e.g. means) or other basic estimates (e.g. regression coefficient) AND variation (e.g. standard deviation) or associated estimates of uncertainty (e.g. confidence intervals)
☐	☒	For null hypothesis testing, the test statistic (e.g. F, t, r) with confidence intervals, effect sizes, degrees of freedom and P value noted<br>*Give P values as exact values whenever suitable.*
☒	☐	For Bayesian analysis, information on the choice of priors and Markov chain Monte Carlo settings
☒	☐	For hierarchical and complex designs, identification of the appropriate level for tests and full reporting of outcomes
☒	☐	Estimates of effect sizes (e.g. Cohen's d, Pearson's r), indicating how they were calculated

Our web collection on statistics for biologists contains articles on many of the points above.

Software and code

Policy information about availability of computer code

Data collection	Labview 2016, BD FACSAria, FACSDiva v9, Gromacs v2019, NAMD2
Data analysis	MATLAB 2020b (Code for analysis in Github (https://github.com/chengzhulab/NCOMMS-22-20167) or available on 10.5281/zenodo.7709746), PyMol 2.3, Microsoft Excel 2021, GraphPad Prism 8, Flowjo v10

For manuscripts utilizing custom algorithms or software that are central to the research but not yet described in published literature, software must be made available to editors and reviewers. We strongly encourage code deposition in a community repository (e.g. GitHub). See the Nature Portfolio guidelines for submitting code & software for further information.

Data

Policy information about availability of data

All manuscripts must include a data availability statement. This statement should provide the following information, where applicable:

- Accession codes, unique identifiers, or web links for publicly available datasets
- A description of any restrictions on data availability
- For clinical datasets or third party data, please ensure that the statement adheres to our policy

All data supporting the findings of this study are included in the article and Supplementary Information. Previously published bond lifetime data4, 5, 6, 8, 9, 13, 16, 28 re-analyzed for model fitting are summarized and deposited in Github (https://github.com/Chengzhulab/NCOMMS-22-20167 or available at Zenodo (ref). PDB structures were used to either apply MD simulation or identify the structural variabilities (end-to-end-/inter- distance of the bound state and the angle between domains) (2C TCR complexed with H2-Kb (PDB codes 2CKB, 1MWA, and 1G6R) and H2-Ldm31 (2E7L), 1G4 TCR complexed with HLA-A2 (2BNR and 2BNQ). P14 TCR

(5M00), NP1-B17 TCR complexed with H2-Db (5SWZ), E8 TCR with HLA-DR (2IAM, 2IAN), 2B4 with I-Ek (6BGA, 3QIB), and TCR-CD3 complex (6JXR). Source data are provided with this paper.

Human research participants

Policy information about studies involving human research participants and Sex and Gender in Research.

Reporting on sex and gender	Gender was not considered because they are irrelevant. We only used human red blood cell (RBC) to assemble the BFP force transducer for force measurement, not to any biological aspects of the RBC or the donor. RBCs from any donor work the same regardless of their gender.
Population characteristics	We used RBCs from healthy male and female (not pregnant) adult donors aged 20 to 40 who weigh at least 110 pounds.
Recruitment	Donors were recruited by word of mount from volunteers from the Georgia Tech community with full consent. Participants were students and staffs on campus.
Ethics oversight	Institutional Review Board of the Georgia Institute of Technology.

Note that full information on the approval of the study protocol must also be provided in the manuscript.

Field-specific reporting

Please select the one below that is the best fit for your research. If you are not sure, read the appropriate sections before making your selection.

[x] Life sciences [ ] Behavioural & social sciences [ ] Ecological, evolutionary & environmental sciences

For a reference copy of the document with all sections, see nature.com/documents/nr-reporting-summary-flat.pdf

Life sciences study design

All studies must disclose on these points even when the disclosure is negative.

Sample size	The sample size question is irrelevant for the modeling part of the study. For the experimental part of the study, sample size is relevant to the bond lifetime vs force data. Each curve includes several hundreds of bond lifetime measurements spanning the entire force range measured from >10 cell-bead pairs. In our experience, several tens of measurements per force bin would be sufficient for the data to be statistically stable. For MD simulations, we increase the sample sizes as much as we can (each 50,000 frames from production phase for data analysis), to ensure the differences between the results of different simulations systems are statistically significant according standard error of the mean.
Data exclusions	For BFP experiments, only clear binding events were used for analysis. in order to extract bond parameters. The profiles of such binding events are detailed in our previous publication (ref. 4, 5). For MD simulations, the data from the equilibration phase were excluded, where the molecules were reshaping under the influences of domain-swapping or external constraints to bring the system to the desired conditions. The stabilized structures and dynamics from the production phase were valid representations of experimental results.
Replication	Replications were not applicable for fitting to the model. All fitting results were determined after checking validity as well as its convergence. For BFP experiments, replications were performed from more than 10 cell(bead)-bead pairs at 3 independent experiments. Each pair had several hundred contacts to see whether there is a bond and how long it can last under force. All attempts at replication were successful. For flow-cytometry, we collected data (~10000 cell per each state) from 4 different states. For MD simulation, all simulations were performed in multiple independent runs. The tested complexes were simulated under various constant forces.
Randomization	For model application, initial values of parameter sets were randomly selected. However, setting boundaries were generally chosen by biophysical guidance. For example, a tilting angle of bond interface as the one of parameter sets has a range [0, 180] (degree) (boundary condition) defined by mathematics. Hence, boundaries were set by using mathematical, biophysical limitation. For BFP experiments, Each experiment used a new fresh batch of cells, and random pairs were selected from a pool of red blood cells, beads, or cells located in the testing chamber. For flow-cytometry, cells were randomly selected and measured by the instrument. For MD simulation, the randomizations were achieved by random initial velocities for each atom sampling from the Maxwell-Boltzmann distribution for each independent run.
Blinding	BFP experiments and flow-cytometry are not relevant to Blinding. The measurements are recorded automatically by the instrument and cannot be biased by the investigator. Modeling are not relevant to Blinding. The fittings are done automatically by computer and cannot be biased by the investigator. MD simulation systems are subject to force fields derived from first principles and self-evolved based on Newton's laws of motion, thus yielding results not biased by human expectations.

Reporting for specific materials, systems and methods

We require information from authors about some types of materials, experimental systems and methods used in many studies. Here, indicate whether each material, system or method listed is relevant to your study. If you are not sure if a list item applies to your research, read the appropriate section before selecting a response.

Materials & experimental systems

n/a	Involved in the study	
☐	☒	Antibodies
☐	☒	Eukaryotic cell lines
☒	☐	Palaeontology and archaeology
☐	☒	Animals and other organisms
☒	☐	Clinical data
☒	☐	Dual use research of concern

Methods

n/a	Involved in the study	
☒	☐	ChIP-seq
☐	☒	Flow cytometry
☒	☐	MRI-based neuroimaging

Antibodies

Antibodies used

The following antibodies used for flow cytometry were bought from BD Biosciences (San Jose, CA): PE-Cy7 anti-mouse CD25 (clone PC61, 1:20, cat. 552880), PE anti-mouse CD69 (clone H1.2F3, 1:20, cat. 553237), APC anti-mouse CD8 (clone 53-6.7, 1:20, cat. 553035) mAbs.
The following antibodies used for flow cytometry were bought from eBioscience (ThermoFisher SCIENTIFIC):
PE anti-mouse CD3ε (clone 145-2C11 or 2C11, cat. 12-0031-82) and allophycocyanin (APC)-conjugated anti-TCRβ (clone H57-597 or H57, cat. 17-5961-82) mAbs.

Validation

PC61, Ceredig, Rhodri, et al. "Expression of interleukin-2 receptors as a differentiation marker on intrathymic stem cells." Nature 314.6006 (1985): 98-100.
H1.2F3, Bendelac, A., et al. "Activation events during thymic selection." The Journal of experimental medicine 175.3 (1992): 731-742.
53-6.7, Bierer BE, Sleckman BP, Ratnofsky SE, Burakoff SJ. The biologic roles of CD2, CD4, and CD8 in T-cell activation. Annu Rev Immunol. 1989; 7:579-599. (Biology)
145-2C11, Setoguchi, Ruka, et al. "Homeostatic maintenance of natural Foxp3+ CD25+ CD4+ regulatory T cells by interleukin (IL)-2 and induction of autoimmune disease by IL-2 neutralization." The Journal of experimental medicine 201.5 (2005): 723-735
H57-597, Ishikawa, Eri, et al. "Protein kinase D regulates positive selection of CD4+ thymocytes through phosphorylation of SHP-1." Nature communications 7.1 (2016): 1-14.

Eukaryotic cell lines

Policy information about cell lines and Sex and Gender in Research

Cell line source(s)	Naive CD8+ mice T cell, Mouse 58-/- T cell hybridoma cells, RBC
Authentication	These cells were widely used to express different TCRs in many publications, including our publications.
Mycoplasma contamination	Mycoplasma contamination was not tested.
Commonly misidentified lines (See ICLAC register)	N/A

Animals and other research organisms

Policy information about studies involving animals; ARRIVE guidelines recommended for reporting animal research, and Sex and Gender in Research

Laboratory animals	C57BL/6J mice (Jackson Laboratories, Strain #000664) were used to generate 2C TCR transgenic mice (6-8 week old male or female mice was used to extract primary T cells from the spleen). Primary T cell purified from the spleen of mice was used for each round of BFP experiment when they were needed.
Wild animals	No wild animals were used in this study.
Reporting on sex	Sex was not considered in this study since we used only primary T cells purified from the spleen of mice.
Field-collected samples	No field samples were used in this study.
Ethics oversight	The Institute Animal Care and Use Committee (IACUC) of Emory University.

Note that full information on the approval of the study protocol must also be provided in the manuscript.

Flow Cytometry

Plots

Confirm that:

- [x] The axis labels state the marker and fluorochrome used (e.g. CD4-FITC).
- [x] The axis scales are clearly visible. Include numbers along axes only for bottom left plot of group (a 'group' is an analysis of identical markers).
- [x] All plots are contour plots with outliers or pseudocolor plots.
- [x] A numerical value for number of cells or percentage (with statistics) is provided.

Methodology

Sample preparation	An aliquot of sample (1 to 5µL depending on cell count) would be added to 100 µL of FACS buffer and antibodies would be added according to dilution factors listed. Sample would be rotated with antibody in the dark for 1 hour. Sample was washed 3X with 500µL of FACS buffer and resuspended in FACS tube with 500uL FACS buffer to be analyzed in flow cytometer.
Instrument	BD FACSAria
Software	FACSDiva v9, Flowjo v10
Cell population abundance	A single population of cells were analyzed to quantify the expression level of TCR, CD8, CD69, and CD25 with single-color staining.
Gating strategy	All cell lines were first gated on FSC/SSC. Gating were based on the expression or coating of molecule of interest.

- [x] Tick this box to confirm that a figure exemplifying the gating strategy is provided in the Supplementary Information.
